# 2,3‐Epoxyamide‐alcohols in Domino Reactions: En Route to Molecular Diversity

**DOI:** 10.1002/open.202400115

**Published:** 2024-05-16

**Authors:** Abderrahman El Bouakher, Jérôme Lhoste, Arnaud Martel, Sébastien Comesse

**Affiliations:** ^1^ Normandie Univ UNILEHAVRE FR 3038 CNRS URCOM 76600 Le Havre France; ^2^ IMMM UMR 6283 CNRS Le Mans Université 72085 Le Mans France

**Keywords:** 2,3-epoxyamido-alcohols, domino reaction, polycyclic lactams, epoxide opening, molecular diversity

## Abstract

The synthesis of polycyclic γ‐ and δ‐lactams bearing up to four contiguous fully controlled stereocenters is presented. For that purpose, we developed an original approach based on the use of 2,3‐epoxyamides in domino reactions by taking advantage of the nucleophilic nitrogen atom and electrophilic epoxide. In reaction with enol ethers bearing gem bis‐electrophiles on the double bond as Michael acceptors, four different reaction pathways were observed. They all started with a domino oxa‐Michael/aza‐Michael/epoxide opening sequence and depending on substrates engaged could be followed either by a lactonization or a hemiketalization/retro‐aldol cascade. Thus, four original fully‐substituted piperidine‐ or pyrrolidine‐2‐one scaffolds were selectively synthesized in good to high yields. Moreover, these polycyclic lactams were obtained in high stereo‐ and chemo‐selectively highlighting the efficiency and molecular diversity offered by this new methodology that should offer various synthetic opportunities in the future.

## Graphical abstract

Molecular diversity was achieved by reaction between easily accessible 2,3‐epoxyamido‐alcohols and Michael acceptors. 4 different γ‐ and δ‐lactam skeletons were selectively synthesized in good yields with up to 4 contiguous stereo‐centres fully controlled. Depending on the R substituent and the electron‐withdrawing groups different domino pathways, all triggered by an an oxa‐Michael/aza‐Michael/epoxide opening sequence, occurred.

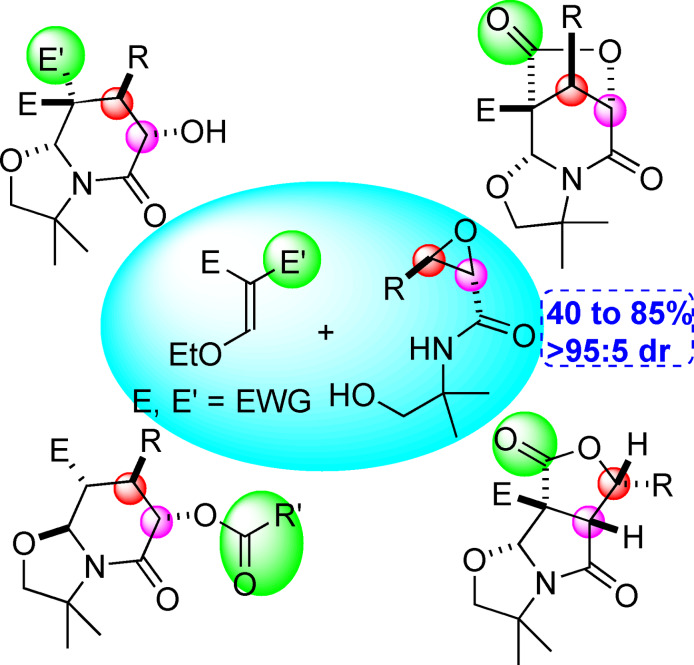

## Introduction

Synthesis of original three‐dimensional structures based on sp^3^ carbons remains an entertaining arena for organic and medicinal chemists as they are often correlated to many compounds with high biological interest.^[1]^ Over the years, many strategies have emerged for the efficient synthesis of such chiral molecules such as the so‐called target‐oriented (TOS) and diversity‐oriented syntheses (DOS).^[2]^ The former's goal is directed by the synthesis of targeted molecules when the latter are aimed to generate a range of structurally different small‐3D‐molecules starting from common starting material. Other powerful tools for the synthesis of three‐dimensional edifices are multicomponent^[3]^ and domino^[4]^ reactions. Another way to achieve this goal can rely on substrate‐controlled^[5]^ oriented synthesis where well‐chosen substituents can lead to molecular diversity by switching domino pathways.

**Scheme 1:**
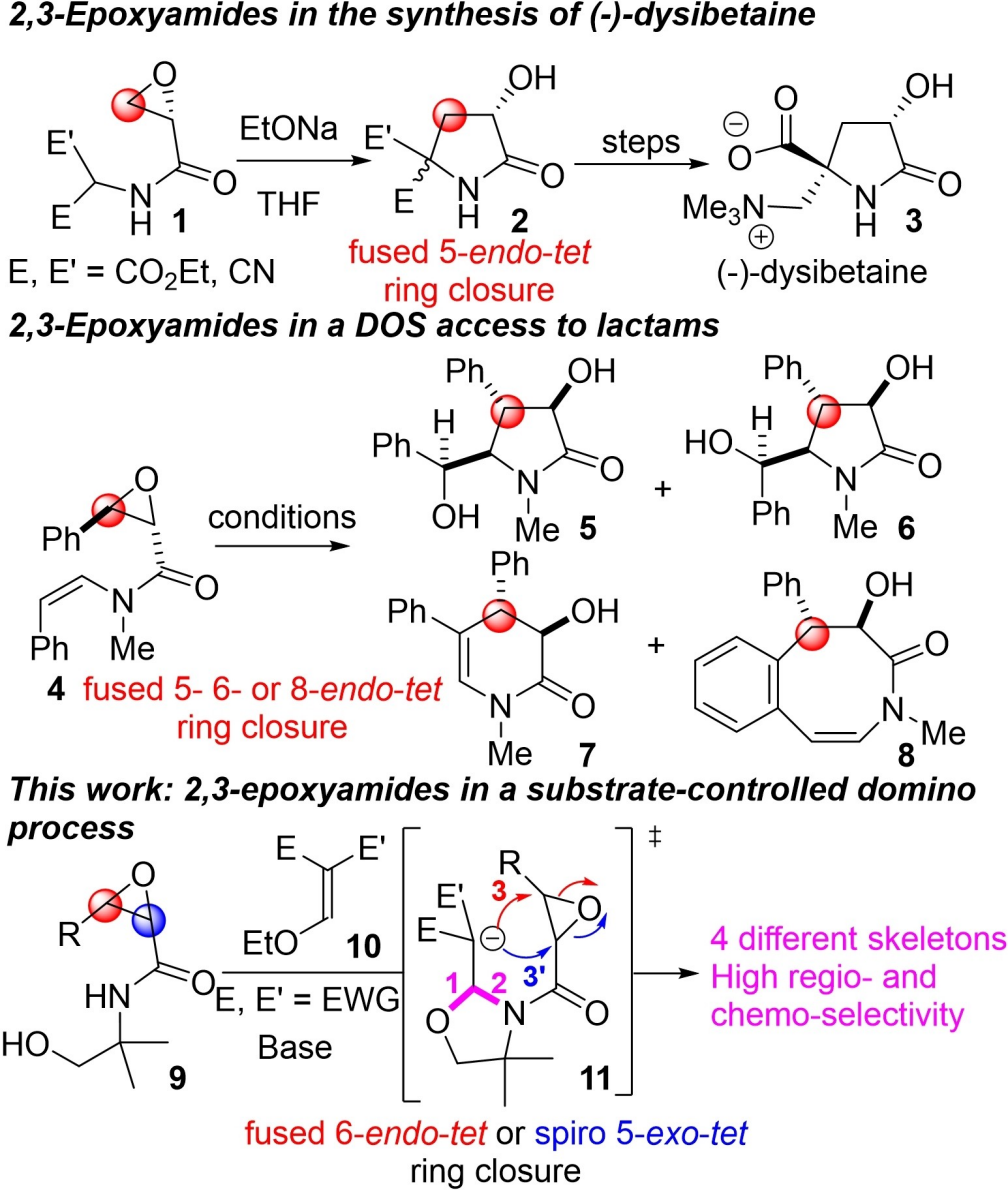
2,3‐Epoxyamides **1**, **4** and **9** as key partners for the synthesis of lactams.

2,3‐Epoxyamides (also named glycidamides or glycidic amides) have several strings to one‘s bow as they are: (i) found in natural products,^[6]^ (ii) parts of molecules exhibiting interesting biological activities^[7]^ and (iii) versatile building blocks bearing multiple reactive centers.^[8]^ For example, Barry B. Snider and Yonghong Gu have demonstrated the synthetic interest of 2,3‐epoxyamides **1** as key intermediates for the total synthesis of (−)‐ and (+)‐dysibetaine **3**, a γ‐lactam isolated from an aqueous extract of the marine sponge *Dysidea herbacea* (upper part of Scheme 1).^[9]^ In their case, only the fused 5‐*endo*‐*tet* ^[10]^ ring closure product **2**, resulting from a carbanion generated in‐situ in α of the electron‐withdrawing groups (EWG) by reaction with EtONa, was observed.

Moreover, as beautifully illustrated by Mei‐Xiang Wang and co‐workers,^[11]^ DOS of multiple lactam rings could be achieved by tuning the reaction conditions (middle part of Scheme 1). Indeed, depending on the additive employed, three different pathways were observed from **4**: (i) a fused 5‐*endo*‐*tet* ring closure probably resulting from the addition of the α‐carbon of the enamide onto the epoxide leading to diastereoisomers **5** and **6**, (ii) a fused 6‐*endo*‐*tet* ring closure based on attack of the β‐carbon of the enamide onto the epoxide forming **7** and (iii) a fused 8‐*endo*‐*tet* ring closure conducting to **8** *via* an intramolecular Friedel–Crafts addition onto the epoxide.^[12]^

Nevertheless, their use as domino partners, particularly by taking advantage of the nucleophilic nitrogen atom, should offer various synthetic opportunities but is to the best of our knowledge not described. This is particularly astonishing since homolog molecules such as haloacetamides^[13]^ and acrylamides^[14]^ are widely used in domino reactions. On this basis, we decided to study 2,3‐epoxyamido‐alcohols **9** as key partners for the direct access to γ‐ and δ‐lactams.

In link with previous findings from our group,^[15]^ we anticipated that 2,3‐epoxyamido‐alcohols **9** could, by reaction with commercially or synthetically available Michael acceptors **10**, lead to various non planar bicyclic γ‐ and δ‐lactams (lower part of Scheme 1). The original domino process was expected to begin with an oxa‐Michael/aza‐Michael sequence (formation of bonds 1 and 2) leading to the anionic intermediate **11** and it was anticipated that, depending on the nature of the substrates, different pathways could occur. Indeed, the R group was expected to influence the intramolecular addition of the carbanion onto the epoxide either *via* a fused 6‐*endo*‐*tet* ring closure (formation of bond 3) or, contrary to the example cited above, a spiro 5‐*exo*‐*tet* ring closure (formation of bond 3’). Moreover, the nature of the EWG could bring even more molecular diversity by allowing subsequent steps such as lactonization, hemiketalization and retro aldol reactions. Finally, we were able to synthetize with a high regio‐ and chemo‐control four different original polycyclic skeletons in a few steps from simple and easily accessible synthons.

## Results and Discussion

Commercially available Michael acceptor **10 a** and 2,3‐epoxyamido alcohols **9 a** were chosen as benchmark substrates (Table 1). Various bases and solvents were selected based on our previous results in related domino processes.^[15]^ The use of 1.2 equivalents of carbonated bases in refluxing acetonitrile proved to be inefficient, the starting materials being fully recovered (entries 1 and 2, Table 1). The same result was observed when shifting to a stronger base, *i. e. t*‐BuOK in THF at room temperature (entry 3, Table 1). Thankfully, 1.2 equivalent of NaH in THF led to the total conversion of the starting materials, but only traces of the desired lactams were isolated (entry 4, Table 1). Considering that the reaction conditions were too harsh and that a catalytic amount of the base should be sufficient, we then modulated the quantity of NaH engaged. The best results were obtained when 0.5 equivalent were used, allowing the formation of the lactams **12 a** and **13 a** in a good 80 % overall yield and a 3 : 7 ratio (entry 8, Table 1). The uncommon bridged tricyclic 5/6/5 system **13 a** ^[16]^ bearing 4 contiguous stereocenters results from a complementary intramolecular lactonization step between the alcoholate resulting from the epoxide opening and one of the ester functions. The ratio was the same with 0.2 or 0.6 equivalent of NaH, only the yields being impacted (compared entries 5–8, Table 1). It is worth noticing that both lactams **12 a** and **13 a**, resulting from a fused 6‐*endo*‐*tet* ring closure, were isolated as a single diastereomer, demonstrating that the newly created stereocenters were fully controlled by the stereochemistry of the epoxide ring.^[15b]^

**Table 1:** Optimization of the reaction conditions.^[a]^

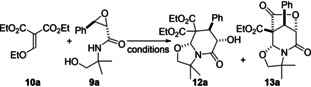					
Entry	Base (equiv.)	Solvent	T °C	Yield (%)^[b]^	Ratio **12 a** : **13 a** ^[c]^
1	K_2_CO_3_ (1.2)	CH_3_CN	reflux	nr^[d]^	–
2	Cs_2_CO_3_ (1.2)	CH_3_CN	reflux	nr^[d]^	–
3	*t*‐BuOK (1.2)	THF	rt	nr^[d]^	–
4	NaH (1.2)	THF	rt	3	nd^[d]^
5	NaH (0.8)	THF	rt	12	nd^[d]^
6	NaH (0.6)	THF	rt	53	3 : 7
7	NaH (0.2)	THF	rt	32	3 : 7
8	NaH (0.5)	THF	rt	80	3 : 7

[a] Base was added to a solution of 2,3‐epoxyamido‐alcohol **9 a** (0.25 mmol, 1 equiv) and Michael acceptor **10 a** (0.26 mmol, 1.05 equiv) in freshly distilled solvent (2 mL). [b] Isolated Yields. [c] dr: diastereomeric ratio determined on the crude mixture. [d] nd: not determined.

**Scheme 2:**
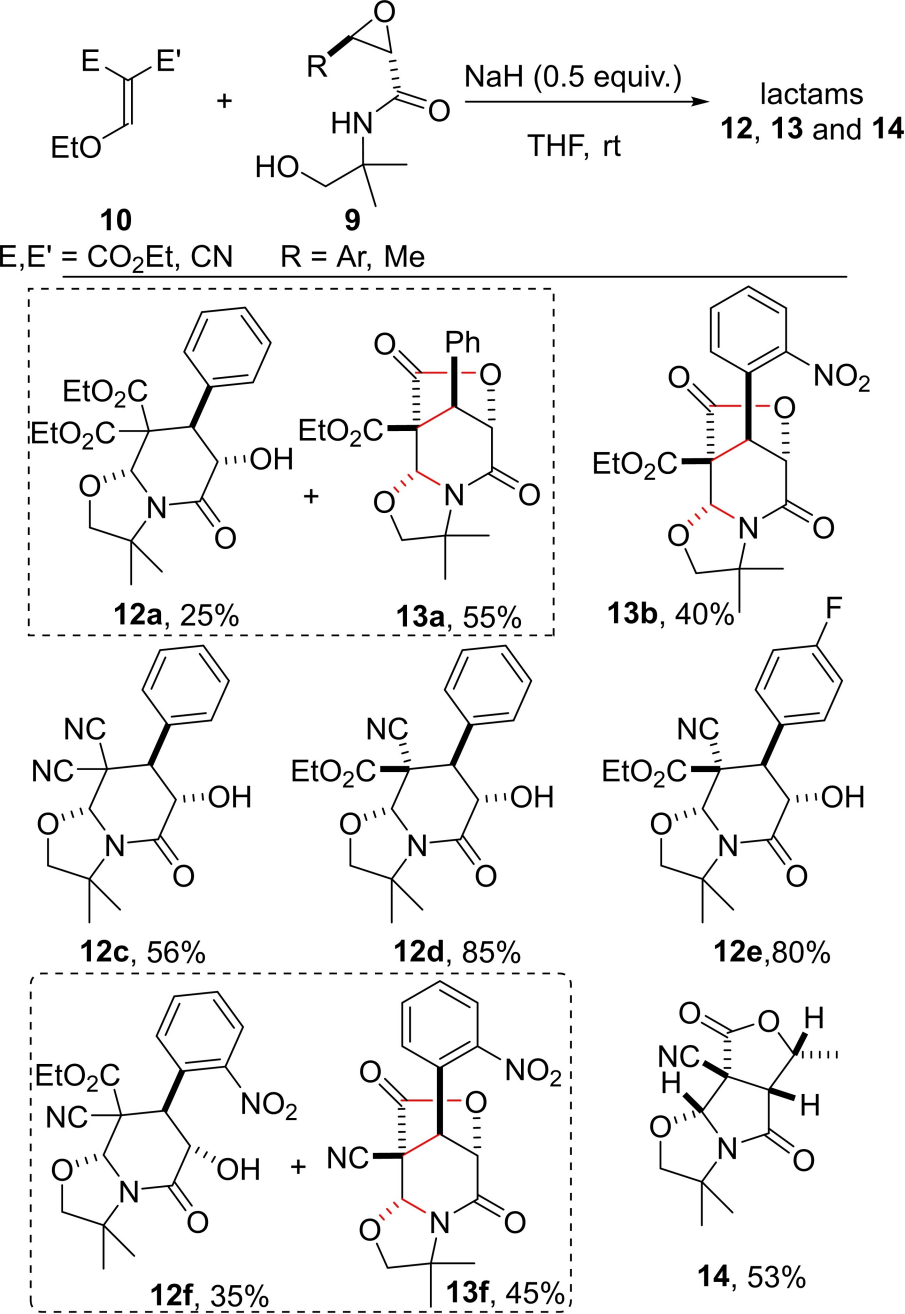
Access to polycyclic lactams **12**, **13** and **14**.

With the optimized conditions in hands, we focused on the substrate scope (Scheme 2). During the reaction between the same Michael acceptor **10 a** (E=E’=CO_2_Et) and 2,3‐epoxyamido alcohols bearing an ortho‐nitro substituent onto the aromatic ring, only the tricyclic lactam **13b** was isolated in 40 % yield. Another symmetrical Michael acceptor **10** (E=E’=CN) was then engaged in the domino process with **9 a** (R=Ph) and the resulting lactam **12 c** was obtained in 56 % yield and >95 : 5 diastereoisomeric ratio. The use of commercially available unsymmetrical enol ether Michael acceptor **10** (E=CN and E’=CO_2_Et) was then considered. Good yields of 85 % and 80 % for lactams **12 d** and **12 e** respectively were secured with all four contiguous stereocenters fully controlled. Surprisingly, with 2,3‐epoxyamido alcohol **9** (R=*o*‐NO_2_−Ph) the course of the domino process was different since a mixture of lactam **12 f** and **13 f** with a total yield of 80 % and a 7 : 9 ratio was obtained. Another different result was observed when replacing the R group onto the 2,3‐epoxyamido alcohol **9** by a methyl substituent (R=Me). Indeed, the new linear tricyclic 5/5/5 skeleton **14** ^[17]^ resulting from the alteration of the domino pathway was isolated in 53 % yield, showing that this time a spiro 5‐*exo*‐*tet* ring closure occurred instead of the fused 6‐*endo*‐*tet* ring closure observed for all the other substrates.

**Scheme 3:**
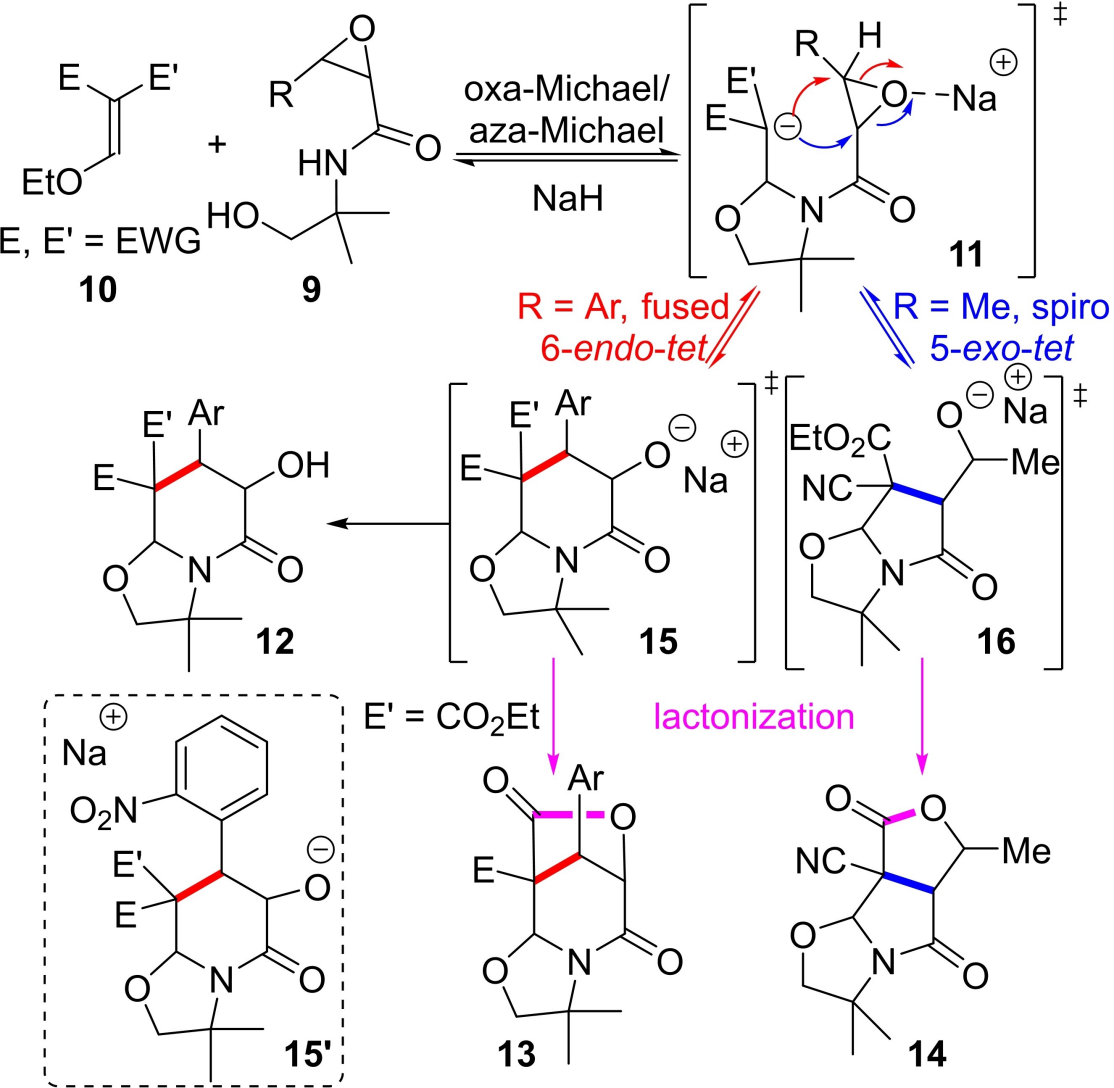
Proposed mechanism's pathways for the access to **12**, **13** and **14**.

All the proposed mechanism paths explaining the above results have in common the 2 first steps, *i. e*. an oxa‐Michael/aza‐Michael sequence triggered by the deprotonation of the alcohol function of **9** by NaH (Scheme 3). The resulting intermediate carbanion **11** can then follow two different paths depending on the R group onto the epoxide ring. In the case of a methyl substituent (R=Me), a spiro 5‐*exo*‐*tet* ring closure occurred leading, after lactonization of alcoholate **16**, to the tricyclic γ‐butyrolactone‐fused γ‐lactam **14**. The aromatic moiety (R=Ar), probably due to the better stabilization of a higher partial positive charge, in the transition state on the carbon bearing this substituent, making it more reactive toward nucleophiles, favor the formation of intermediate **15** *via* a fused 6‐*endo*‐*tet* ring closure in accordance with Mei‐Xiang Wang and co‐workers’ findings (see middle part of Scheme 1). The bicyclic alcoholate **15** could furnish **12** after protonation, either during the process due to a default of base, or during work‐up. It could also afford the tricyclic 5/6/5 lactone **13** when at least one of the electron‐withdrawing groups is an ester function being on the same face of the alcoholate. The peculiar formation of the tricyclic 5/6/5 lactone **13 f**, implying that in this case the ester function was on the same side than the alcoholate contrary to **12 d** and **12 e** (compare stereochemistry of the lactams **12 d**, **12 e** and **13 f**, Scheme 2), could be explained by the complexation of the sodium atom by the *o*‐NO_2_ substituent^[15c]^ in **15’** (Scheme 3) changing the otherwise *trans* selectivity between the ester and the alcoholate.

**Scheme 4:**
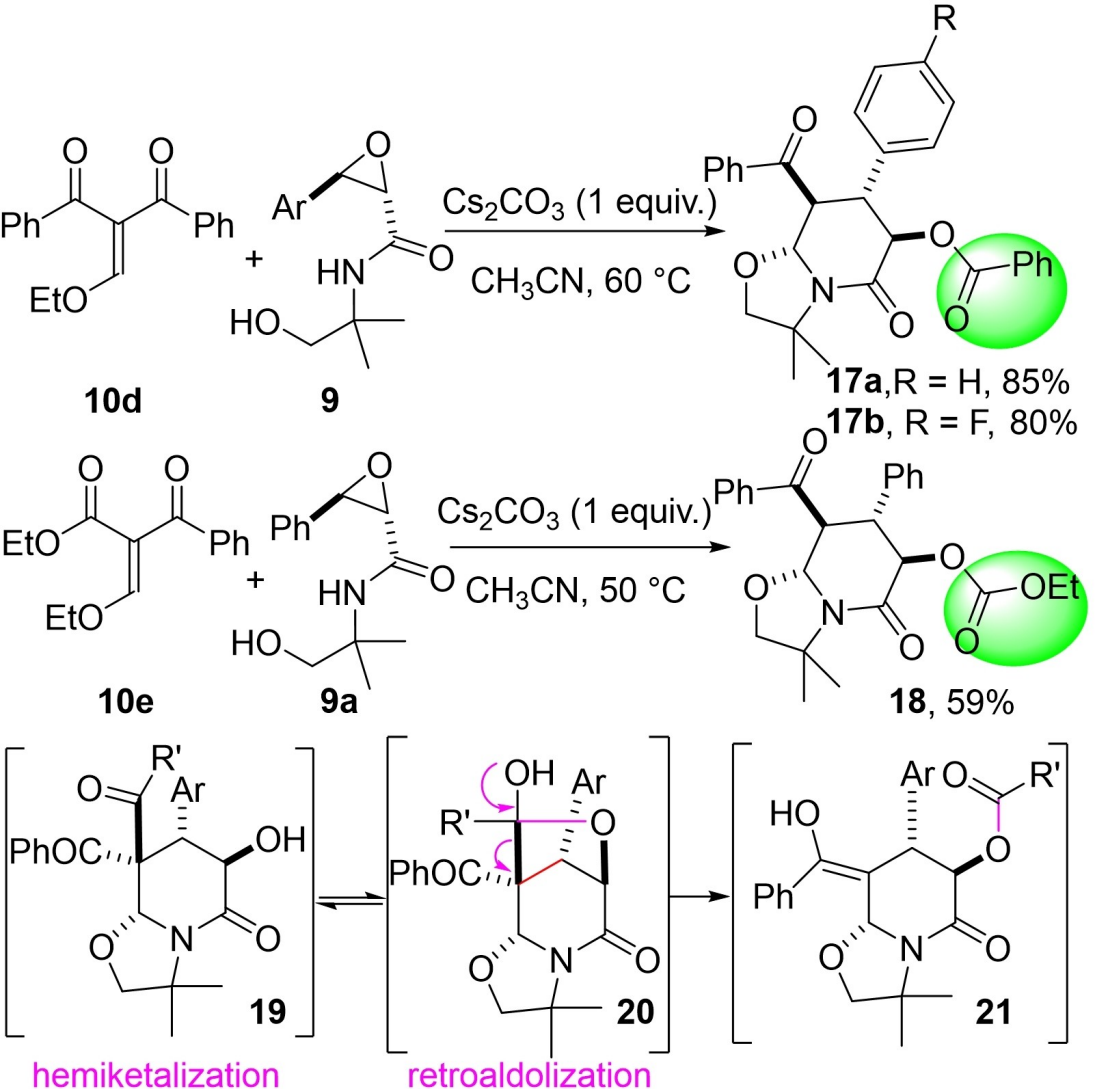
Unexpected rearrangement products **17** and **18** and proposed mechanism.

Hence, we turned our attention to Michael acceptor **10 d** bearing two benzoyl moieties (Scheme 4). The experimental conditions used above proved to be detrimental to the process leading to a complex mixture. Thus, 1 equivalent of cesium carbonate (Cs_2_CO_3_)^[15a]^ as a base in freshly distilled CH_3_CN at 60 or 50 °C were employed (for more details see SI). The course of the domino process changes dramatically to lead to rearranged lactams **17 a** and **17 b** in 85 % and 80 % yield and high dr, respectively.^[18]^ More surprising, in the case of Michael acceptor **10 e** bearing a benzoyl and an ester function, it was the latter which was fully transferred to furnish the totally unexpected bicyclic lactam **18** in 59 % yield with no trace of another diastereoisomer. It is important to notice that we didn't observe the formation of a bridged tricyclic lactone like products **13** obtained above (Scheme 2). An oxa‐Michael/aza‐Michael would first lead to the intermediate carbanion **11** as depicted in Scheme 3. Then, after a fused 6‐*endo*‐*tet* ring closure the bicyclic alcohol **19** could evolve toward the bridged intermediate **20** after an intramolecular hemiketalization. The last step is a retro‐aldol mechanism^[20]^ leading to the formation of the enol intermediate **21**. After a diastereoselective keto‐enol tautomerism favoring a pseudo‐equatorial position for all the substituent onto the piperidin‐2‐one rings, the rearranged products **17 a,b** and **18** are formed. The reactivity shift explaining these surprising results barely disclosed before^[19]^ could be rationalized both by the use of less basic conditions (Cs_2_CO_3_ instead of NaH) favoring the formation of protic intermediates and of higher temperatures (50 or 60 °C vs rt) favoring such rearrangements. These mild basic conditions allow to isolate the rearrangement product without further side‐reactions. It is also assumed that the presence of traces of hydrogen carbonates plays an important role in the various proton transfers involved during this rearrangement.

**Figure 1:**
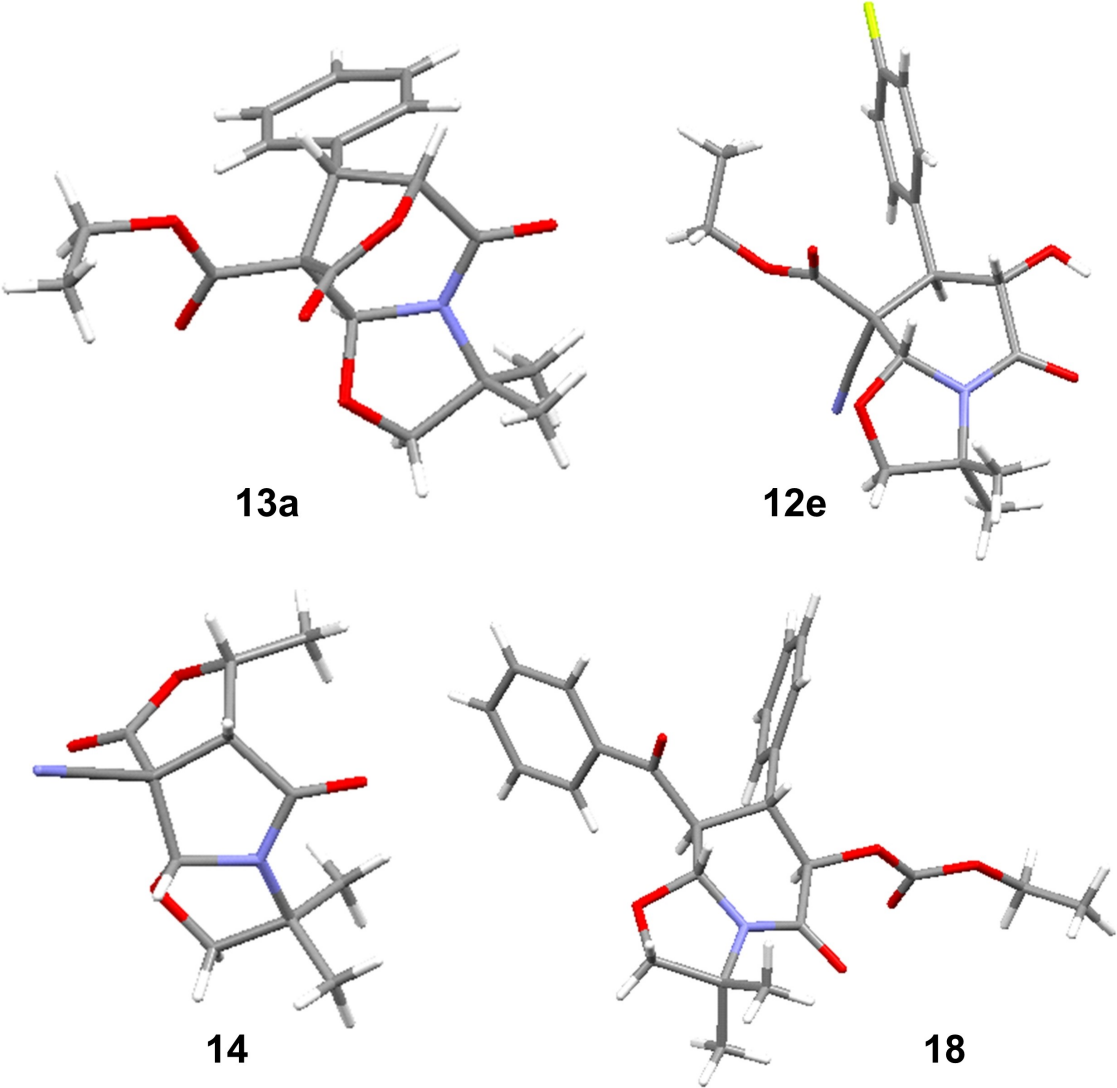
X‐ray structures of domino products **13 a**, **12 e**, **14** and **18**.

The structure and relative stereochemistry of products **13 a**, **12 e**, **14** and **18** were secured by X‐ray analysis and the relative stereochemistry of other lactams extrapolated from them (see SI and Figure 1).^[21]^ For the tricyclic lactone **13 a** a *syn* relationship between the phenyl and ester moieties is observed. The same *syn* relationship between the aromatic ring and the ester on one hand and the hydroxyl and nitrile function on the other hand is shown for the bicyclic lactam **12 e**. The X‐ray structure of the tricyclic 5/5/5 lactone **14** displays that the nitrile moiety and the H atoms onto the three other asymmetric carbons are on the same face. An all trans relationship between adjacent substituents onto lactam **18** is observed. Finally, in all cases, the 4 contiguous stereocenters including the 2 created during the domino process were fully controlled.

## Conclusions

An original access to polycyclic γ‐ and δ‐lactams based on substrate‐controlled oriented synthesis was presented. The key partners of the divergent domino processes were 2,3‐epoxyamido‐alcohols and Michael acceptors. The main finding of this study was the possibility to fully switch the epoxide opening in order to access 5‐ or 6‐membered lactam rings. Moreover, the concomitant formation of tricyclic lactones was observed when at least one of the EWG was an ester function or the transfer of one of the EWG onto the alkoxide formed after the epoxide opening could occur. Whatever the domino sequence, good yields and high diastereoselectivity were achieved. We are now working on expanding the scope of these new domino sequences to other Michael acceptors and chiral 2,3‐epoxyamido‐alcohols. Moreover, DFT calculations to elucidate and rationalize the role of the substrate substituents are underway and will be published in due course.

## Supporting Information

The authors have cited additional references within the Supporting Information^[22]^

2,3‐Epoxyamido‐alcohol **9** (0.25 mmol) and Michael acceptor **10** (0.26 mmol, 1.05 eq) were dissolved in freshly distilled THF (2 mL). Sodium hydride (3 mg, 0.125 mmol, 0.5 eq.) was then added. The mixture was stirred for 12 to 48 hours and was then quenched carefully at 0 °C by addition of a saturated aqueous solution of NH_4_Cl (2 mL). The aqueous layer was extracted with EtOAc (3×5 mL), the organic layers were combined, brined, dried over MgSO_4_ and the solvent was removed under vacuum. The residue was then chromatographed on silica gel to provide the desired domino compound.

Experimental procedures, characterization data, and copy of the^1^H and ^13^C NMR spectra for all new compounds (**9**, **12**–**14, 17** and **18**).

## Conflict of Interests

The authors declare no conflict of interest.

1

## Supporting information

As a service to our authors and readers, this journal provides supporting information supplied by the authors. Such materials are peer reviewed and may be re‐organized for online delivery, but are not copy‐edited or typeset. Technical support issues arising from supporting information (other than missing files) should be addressed to the authors.

Supporting Information

## Data Availability

The data that support the findings of this study are available in the supplementary material of this article.
