# Supplementary material for: 2,3‐Epoxyamide‐alcohols in Domino Reactions: En Route to Molecular Diversity
Source: ChemistryOpen. 2024 May 16;13(9):e202400115. doi: 10.1002/open.202400115 (PMC11977405; doi:10.1002/open.202400115)

ChemistryOpen

Supporting Information

2,3-Epoxyamide-alcohols in Domino Reactions: En Route to Molecular Diversity

Abderrahman El Bouakher, Jérôme Lhoste, Arnaud Martel, and Sébastien Comesse*

Supporting Information

2,3-Epoxyamido-alcohols in Domino Reactions: en Route to Molecular Diversity

Abderrahman El Bouakher,† Jérôme Lhoste,‡ Arnaud Martel‡ and Sébastien Comesse*,†

†Normandie Univ, UNILEHAVRE FR 3038 CNRS, URCOM 76600 Le Havre, France
‡IMMM, UMR 6283 CNRS, Le Mans Université, 72085 Le Mans, France

Table of Contents

General Considerations 2
General scheme and procedure for preparation of 2,3-epoxyamido-alcohols 9 2
Procedures for the synthesis of polycyclic lactams 4
X-ray Crystal Structure Determination of compounds 13a, 12e, 14 and 18 9
^{1}H and ^{13}C NMR spectra 15

1. **General Considerations**

All commercially available starting materials have been used without further purification. Compositions of stereoisomeric mixtures were determined by ^{1}H NMR analysis of the crude mixture before any purification. Melting points (Mp) were taken with a SMP10 capillary melting point apparatus (Stuart) and are uncorrected. FT-IR spectra were recorded with a Perkin-Elmer Frontier. The NMR spectra were recorded on a 300 UltraShield instrument (Bruker) as solutions in $CDCl_3$ at 300 MHz (^{1}H) and 75 MHz (^{13}C), respectively, and chemical shifts (δ) are expressed in ppm. High resolution mass spectra were recorded on a 6530 Q-TOF (Agilent Technologies). Thin layer chromatography (TLC) was performed using silica gel analytical plates (*F*254) of 0.25 mm thickness. The detection on TLC plates was performed by UV light at 254 or 365 nm or using a permanganate revelator.

2. **General scheme and procedure for preparation of 2,3-epoxyamido-alcohols 9:**

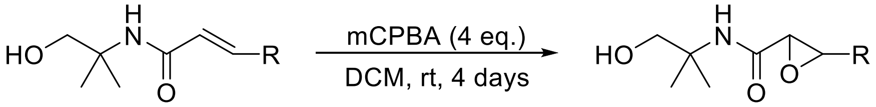

Scheme 1. Synthesis of 2,3- epoxyamido-alcohols **9**

General procedure: The required acrylamide (6.36 mmol) was dissolved in DCM (15 mL). *m*CPBA (25.44 mmol, 4 eq.) was then added at room temperature. The mixture was stirred for 4 days and was then quenched by addition of a saturated aqueous solution of $NaHCO_3$ (15 mL). The aqueous layer was extracted with DCM (3 × 15 mL), the organic layers were combined, brine, dried over $MgSO_4$ and solvent was removed under vacuum. The residue was then chromatographed on silica gel to provide the desired compound.

***N*-(1-Hydroxy-2-methylpropan-2-yl)-3-phenyloxirane-2-carboxamide 9a:**
The crude material was purified by flash chromatography on silica gel (eluting with Cyclohexane/EtOAc = 50:50) to give the title compound as a white solid. Yield: 80%.

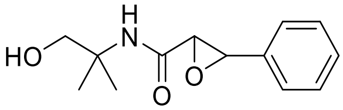

Rf = 0.475 (cyclohexane/EtOAc: 50/50); mp. 140 °C; ^{1}**H NMR** (300 MHz, $CDCl_3$) δ 1.33 (s, 3H), 1.34 (s, 3H), 3.49 (d, *J* = 2.0 Hz, 1H), 3.62 (d, *J* = 12.3 Hz, 1H), 3.66 (d, *J* = 12.3 Hz, 1H), 3.88 (d, *J* = 2.0 Hz, 1H), 4.44 (s, 1H), 6.32 (s, 1H), 7.26-7.30 (m, 2H), 7.35-7.40 (m, 3H) ppm; ^{13}**C NMR** (75 MHz, $CDCl_3$) δ 24.5, 24.6, 56.0, 59.0, 59.3, 70.0, 125.8 (2 x CH), 128.7 (2 x CH), 129.1, 134.7, 168.0 ppm; **IR** (neat): υ 3354, 3285, 1644, 1543, 1057, 1022, 885, 817, 753 cm^{-1}; **HRMS (EI)** m/z: calculated for $C_{13}H_{18}NO_3$ $[M+H]^+$: 236.1287, found: 236.1292.

N-(1-Hydroxy-2-methylpropan-2-yl)-3-(2-nitrophenyl)oxirane-2-carboxamide 9b:

The crude material was purified by flash chromatography on silica gel (eluting with Cyclohexane/EtOAc = 50:50) to give the title compound as a yellow solid. Yield: 40%.

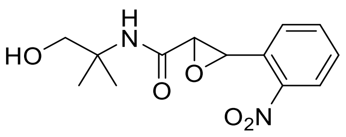

Rf = 0.25 (cyclohexane/EtOAc: 50/50); mp. 127 °C; **^{1}H NMR** (300 MHz, $CDCl_3$) δ 1.36 (s, 6H), 3.28 (s, 1H), 3.37 (d, *J* = 2.1 Hz, 1H), 3.65 (d, *J* = 11.7 Hz, 1H), 3.70 (d, *J* = 11.8 Hz, 1H), 4.56 (d, *J* = 1.7 Hz, 1H), 6.26 (s, 1H), 7.53-7.59 (m, 2H), 7.71 (dd, *J* = 10.9, 4.1 Hz, 1H), 8.21 (d, *J* = 8.4 Hz, 1H) ppm; **^{13}C NMR** (75 MHz, $CDCl_3$) δ 24.5, 24.6, 56.2, 57.0, 58.3, 69.8, 125.0, 126.9, 129.5, 131.7, 134.5, 147.6, 167.1 ppm; **IR** (neat): υ 3284, 1661, 154, 1525, 1339, 1060, 792, 739 cm^{-1}; **HRMS (EI)** m/z: calculated for $C_{13}H_{16}N_2O_5Na$ $[M+Na]^+$: 303.0957, found: 303.0964.

3-(4-Fluorophenyl)-N-(1-hydroxy-2-methylpropan-2-yl)oxirane-2-carboxamide 9c:

The crude material was purified by flash chromatography on silica gel (eluting with Cyclohexane/EtOAc = 50:50) to give the title compound as a white solid. Yield: 65%.

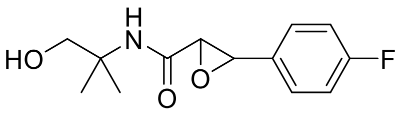

Rf = 0.375 (cyclohexane/EtOAc: 50/50); mp. 94 °C; **^{1}H NMR** (300 MHz, $CDCl_3$) δ 1.33 (s, 3H), 1.34 (s, 3H), 3.45 (d, *J* = 2.0 Hz, 1H), 3.61 (d, *J* = 12.2 Hz, 1H), 3.65 (d, *J* = 12.2 Hz, 1H), 3.86 (d, *J* = 1.9 Hz, 1H), 4.35 (s, 1H), 6.29 (s, 1H), 7.03-7.09 (m, 2H), 7.23-7.28 (m, 2H) ppm; **^{13}C NMR** (75 MHz, $CDCl_3$) δ 24.5, 24.5, 56.0, 58.7, 59.0, 69.9, 115.8 (d, *J* = 21.9 Hz), 127.6 (d, *J* = 8.4 Hz), 130.5 (d, *J* = 3.1 Hz), 163.2 (d, *J* = 248.2 Hz), 167.7 ppm; **IR** (neat): υ 3285, 1643, 1542, 1514, 1224, 1053, 817, 791 cm^{-1}; **HRMS (EI)** m/z: calculated for $C_{13}H_{17}FNO_3$ $[M+H]^+$: 254.1192, found: 254.1197.

N-(1-Hydroxy-2-methylpropan-2-yl)-3-methyloxirane-2-carboxamide 9d:

The crude material was purified by flash chromatography on silica gel (eluting with Cyclohexane/EtOAc = 50:50) to give the title compound as colorless oil. Yield: 70%.

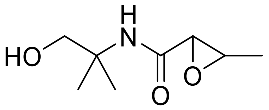

Rf = 0.3 (cyclohexane/EtOAc: 50/50); **^{1}H NMR** (300 MHz, $CDCl_3$) δ 1.19 (s, 3H), 1.20 (s, 3H), 1.31 (d, *J* = 5.2 Hz, 3H), 2.95 (qd, *J* = 5.1, 2.1 Hz, 1H), 3.05 (d, *J* = 2.1 Hz, 1H), 3.45 (d, *J* = 12.1 Hz, 1H), 3.49 (d, *J* = 12.0 Hz, 1H), 6.25 (s, 1H) ppm; **^{13}C NMR** (75 MHz, $CDCl_3$) δ 17.3, 24.0, 24.1, 55.4, 55.8, 56.3, 69.5, 169.0 ppm; **IR** (neat): υ 3365, 1655, 1531, 1057, 862, 772 cm^{-1}; **HRMS (EI)** m/z: calculated for $C_8H_{16}NO_3$ $[M+H]^+$: 174.1128, found: 174.1130.

3. **Procedures for the synthesis of polycyclic lactams:**

Synthesis of diethyl 6-hydroxy-3,3-dimethyl-5-oxo-7-phenyltetrahydro-5*H*-oxazolo[3,2-*a*]pyridine-8,8(8a*H*)-dicarboxylate 12a and ethyl 3,3-dimethyl-5,8-dioxo-10-phenyltetrahydro-8*H*-6,9-methanooxazolo[3,2-*d*][1,4]oxazepine-9(9a*H*)-carboxylate 13a: *N*-(1-hydroxy-2-methylpropan-2-yl)-3-phenyloxirane-2-carboxamide **9a** (59 mg, 0.25 mmol) and diethyl 2-(ethoxymethylene)malonate **10a** (57 mg, 0.26 mmol, 1.05 eq) were dissolved in freshly distilled THF (2 mL). Sodium hydride (3 mg, 0.125 mmol, 0.5 eq.) was then added. The mixture was stirred for 48 hours and was then quenched carefully at 0 °C by addition of a saturated aqueous solution of NH_4Cl (2 mL). The aqueous layer was extracted with EtOAc (3 × 5 mL), the organic layers were combined, brine, dried over $MgSO_4$ and solvent was removed under vacuum. The residue was then chromatographed on silica gel to provide the desired compound.

The crude material was purified by flash chromatography on silica gel (eluting with Cyclohexane/EtOAc = 70:30) to give the title compound as colorless oil. Yield: 25%.

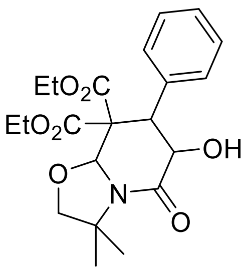

Rf = 0.15 (cyclohexane/EtOAc: 70/30); **^{1}H NMR** (300 MHz, $CDCl_3$) δ 0.82 (t, *J* = 7.2 Hz, 3H), 1.27 (s, 1H), 1.30 (t, *J* = 7.2 Hz, 3H), 1.46 (s, 3H), 1.60 (s, 3H),3.31-3.37 (m, 1H), 3.76-3.83 (m, 1H), 3.83 (d, *J* = 8.0 Hz, 1H), 3.91 (d, *J* = 8.0 Hz, 1H), 3.95 (d, *J* = 9.3 Hz, 1H), 4.16-4.27 (m, 1H), 4.32-4.43 (m, 1H), 4.38 (d, *J* = 9.4 Hz, 1H), 5.91 (s, 1H), 7.21-7.24 (m, 2H), 7.29-7.38 (m, 3H) ppm; **^{13}C NMR** (75 MHz, $CDCl_3$) δ 13.3, 14.0, 23.3, 24.0, 51.4, 60.3, 61.5, 62.5, 63.0, 70.9, 79.7, 87.6, 127.7 (2 x CH), 128.5 (2 x CH), 129.0, 138.7, 167.4, 167.5, 168.3 ppm; **IR** (neat): υ 3415, 1717, 1668, 1259, 1216, 1093, 1001, 722 cm^{-1}; **HRMS (EI)** m/z: calculated for $C_{21}H_{28}NO_7$ $[M+H]^+$: 406.1866, found: 406.1877.

The crude material was purified by flash chromatography on silica gel (eluting with Cyclohexane/EtOAc = 70:30) to give the title compound as a white solid. Yield: 55%.

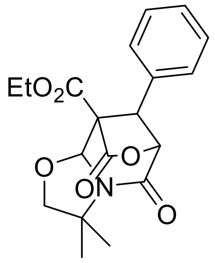

Rf = 0.625 (cyclohexane/EtOAc: 70/30); mp. 149 °C; **^{1}H NMR** (300 MHz, $CDCl_3$) δ 1.38 (s, 3H), 1.42 (d, *J* = 7.1 Hz, 3H), 1.51 (s, 3H), 3.54 (d, *J* = 8.9 Hz, 1H), 3.92 (d, *J* = 8.9 Hz, 1H), 4.41-4.52 (m, 2H), 4.55 (d, *J* = 4.9 Hz, 1H), 4.77 (s, 1H), 5.02 (d, *J* = 4.9 Hz, 1H), 7.12-7.14 (m, 2H), 7.39-7.41 (m, 3H) ppm; **^{13}C NMR** (75 MHz, $CDCl_3$) δ 14.1, 23.6, 24.0, 51.1, 58.4, 60.1, 63.0, 78.6, 79.0, 84.6, 126.9 (2 x CH), 129.1, 129.4 (2 x CH), 130.0, 163.4, 165.9, 168.3 ppm; **IR** (neat): υ 1806, 1743, 1684, 1436, 1244, 1084, 1037, 1000, 740 cm^{-1}; **HRMS (EI)** m/z: calculated for $C_{19}H_{22}NO_6$ $[M+H]^+$: 360.1447, found: 360.1435.

Synthesis of ethyl 3,3-dimethyl-10-(2-nitrophenyl)-5,8-dioxotetrahydro-8*H*-6,9-methanooxazolo[3,2-*d*][1,4]oxazepine-9(9a*H*)-carboxylate 13b: *N*-(1-Hydroxy-2-methylpropan-2-yl)-3-(2-nitrophenyl)oxirane-2-carboxamide **9b** (70 mg, 0.25 mmol) and diethyl 2-(ethoxymethylene)malonate **10a** (57 mg, 0.26 mmol, 1.1 eq) were dissolved in

freshly distilled THF (2 mL). Sodium hydride (3 mg, 0.125 mmol, 0.5 eq.) was then added. The mixture was stirred for 12 hours and was then quenched carefully at 0 °C by addition of a saturated aqueous solution of NH_4Cl (2 mL). The aqueous layer was extracted with EtOAc (3 × 5 mL), the organic layers were combined, brine, dried over $MgSO_4$ and solvent was removed under vacuum. The residue was then chromatographed on silica gel to provide the desired compound

The crude material was purified by flash chromatography on silica gel (eluting with Cyclohexane/EtOAc = 70:30) to give the title compound as a yellow solid. Yield: 40%.

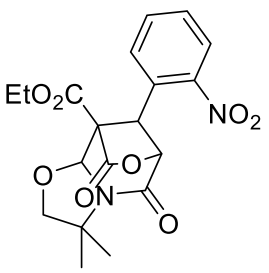

Rf = 0.225 (cyclohexane/EtOAc: 70/30); mp. 165 °C; **^{1}H NMR** (300 MHz, $CDCl_3$) δ 1.19 (s, 3H), 1.29 (s, 3H), 1.41 (t, *J* = 7.1 Hz, 3H), 3.86 (d, *J* = 8.7 Hz, 1H), 3.98 (d, *J* = 8.7 Hz, 1H), 4.43 (qd, *J* = 7.1, 2.6 Hz, 2H), 4.50 (d, *J* = 8.1 Hz, 1H), 5.78 (s, 1H), 6.60 (d, *J* = 8.0 Hz, 1H), 7.55-7.61 (m, 1H), 7.70 (dd, *J* = 3.9, 1.1 Hz, 2H), 8.33 (d, *J* = 8.1 Hz, 1H) ppm; **^{13}C NMR** (75 MHz, $CDCl_3$) δ 14.0, 23.4, 25.3, 55.0, 57.7, 59.5, 63.7, 78.1, 82.6, 91.0, 125.3, 126.8, 129.5, 131.6, 134.0, 147.1, 165.8, 165.9, 166.8 ppm; **IR** (neat): υ 1789, 1746, 1702, 1527, 1335, 1243, 1147, 994, 733 cm^{-1}; **HRMS (EI)** m/z: calculated for $C_{19}H_{21}N_2O_8$ $[M+H]^+$: 405.1298, found: 405.1306.

Synthesis of 6-hydroxy-3,3-dimethyl-5-oxo-7-phenyltetrahydro-5*H*-oxazolo[3,2-*a*]pyridine-8,8(8a*H*)-dicarbonitrile 12c: *N*-(1-hydroxy-2-methylpropan-2-yl)-3-phenyloxirane-2-carboxamide **9a** (59 mg, 0.25mmol), 2-(ethoxymethylene)malononitrile **10b** (45 mg, 0.375 mmol, 1.5 eq) were dissolved in freshly distilled THF (2 mL). Sodium hydride (3 mg, 0.125 mmol, 0.5 eq.) was then added. The resulting mixture was stirred for 12 hours and was then quenched by addition of a saturated aqueous solution of NH_4Cl (5 mL). The aqueous layer was extracted with EtOAc (3 × 5 mL), the organic layers were combined, brine, dried over $MgSO_4$ and evaporated. The residue was then chromatographed on silica gel to give the desired compound.
The crude material was purified by flash chromatography on silica gel (eluting with Cyclohexane/EtOAc = 70:30) to give the title compound as a yellow solid. Yield: 56%.

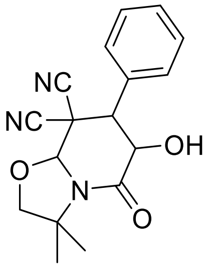

Rf = 0.125 (cyclohexane/EtOAc: 70/30); mp. 235 °C; **^{1}H NMR** (300 MHz, $CDCl_3$) δ 1.63 (s, 3H), 1.67 (s, 3H), 1.40 (bs, 1H), 3.46 (d, *J* = 11.0 Hz, 1H), 3.92 (d, *J* = 8.7 Hz, 1H), 4.17 (d, *J* = 8.7 Hz, 1H), 4.69 (d, *J* = 11.0 Hz, 1H), 5.34 (s, 1H), 7.49-7.55 (m, 5H) ppm; **^{13}C NMR** (75 MHz, $CDCl_3$) δ 23.2, 23.9, 44.5, 49.2, 61.7, 69.4, 79.9, 89.0, 110.3, 110.8, 128.2 (2 x CH), 129.5 (2 x CH), 130.0, 132.2, 166.4 ppm; **IR** (neat): υ 3400, 1648, 1435, 1304, 1090, 1074, 720 cm^{-1}; **HRMS (EI)** m/z: calculated for $C_{17}H_{18}N_3O_3$ $[M+H]^+$: 312.1348, found: 312.1355.

Synthesis of ethyl 8-cyano-6-hydroxy-3,3-dimethyl-5-oxo-7-phenylhexahydro-5*H*-oxazolo[3,2-*a*]pyridine-8-carboxylate 12d: *N*-(1-hydroxy-2-methylpropan-2-yl)-3-phenyloxirane-2-carboxamide **9a** (59 mg, 0.25 mmol) and (*E*)-ethyl 2-cyano-3-ethoxyacrylate

10c (44.4 mg, 0.26 mmol, 1.1 eq) were dissolved in freshly distilled THF (2 mL). Sodium hydride (3 mg, 0.125 mmol, 0.5 eq.) was then added. The mixture was stirred for 12 hours and was then quenched carefully at 0 °C by addition of a saturated aqueous solution of NH_4Cl (2 mL). The aqueous layer was extracted with EtOAc (3 × 5 mL), the organic layers were combined, brine, dried over $MgSO_4$ and solvent was removed under vacuum. The residue was then chromatographed on silica gel to provide the desired compound.
The crude material was purified by flash chromatography on silica gel (eluting with Cyclohexane/EtOAc = 70:30) to give the title compound as a white solid. Yield: 85%.

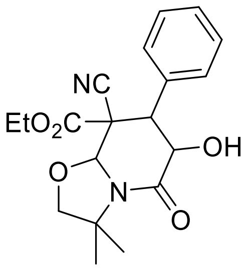

Rf = 0.25 (cyclohexane/EtOAc: 70/30); mp. 169 °C; **^{1}H NMR** (300 MHz, $CDCl_3$) δ 0.85 (t, *J* = 7.2 Hz, 3H), 1.64 (s, 3H), 1.73 (s, 3H), 3.37-3.68 (m, 1H), 3.64 (d, *J* = 11.3 Hz, 1H), 3.76-3.84 (m, 1H), 3.92 (d, *J* = 8.7 Hz, 1H), 3.94 (s, 1H), 4.23 (d, *J* = 8.6 Hz, 1H), 4.65 (d, *J* = 11.5 Hz, 1H), 5.78 (s, 1H), 7.31-7.42 (m, 5H) ppm; **^{13}C NMR** (75 MHz, $CDCl_3$) δ 13.3, 23.5, 24.0, 54.6, 55.3, 60.8, 63.5, 67.3, 80.3, 87.1, 117.1, 128.6 (2 x CH), 128.7, 129.3 (2 x CH), 133.5, 164.4, 167.6 ppm; **IR** (neat): υ 3401, 1740, 1684, 1335, 1238, 1196, 1099, 943, 748, 699 cm^{-1}; **HRMS (EI)** m/z: calculated for $C_{19}H_{23}N_2O_5$ $[M+H]^+$: 359.1607, found: 359.1606.

Synthesis of ethyl 8-cyano-7-(4-fluorophenyl)-6-hydroxy-3,3-dimethyl-5-oxohexahydro-5*H*-oxazolo[3,2-*a*]pyridine-8-carboxylate 12e: 3-(4-fluorophenyl)-*N*-(1-hydroxy-2-methylpropan-2-yl)oxirane-2-carboxamide **9c** (63.3 mg, 0.25 mmol) and (*E*)-ethyl 2-cyano-3-ethoxyacrylate **10c** (44 mg, 0.26 mmol, 1.1 eq) were dissolved in freshly distilled THF (2 mL). Sodium hydride (3 mg, 0.125 mmol, 0.5 eq.) was then added. The mixture was stirred for 12 hours and was then quenched carefully at 0 °C by addition of a saturated aqueous solution of NH_4Cl (2 mL). The aqueous layer was extracted with EtOAc (3 × 5 mL), the organic layers were combined, brine, dried over $MgSO_4$ and solvent was removed under vacuum. The residue was then chromatographed on silica gel to provide the desired compound

The crude material was purified by flash chromatography on silica gel (eluting with Cyclohexane/EtOAc = 70:30) to give the title compound as a white solid. Yield: 80%.

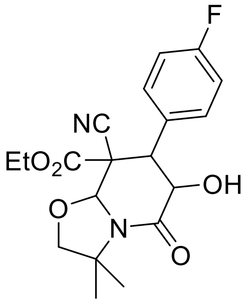

Rf = 0.225 (cyclohexane/EtOAc: 70/30); mp. 194 °C; **^{1}H NMR** (300 MHz, $CDCl_3$) δ 0.92 (t, *J* = 7.2 Hz, 3H), 1.64 (s, 3H), 1.72 (s, 3H), 3.61 (d, *J* = 11.6 Hz, 1H), 3.63-3.76 (m, 1H), 3.82-3.90 (m, 1H), 3.92 (d, *J* = 8.7 Hz, 1H), 3.95 (s, 1H), 4.24 (d, *J* = 8.6 Hz, 1H), 4.60 (d, *J* = 11.7 Hz, 1H), 5.76 (s, 1H), 7.05-7.13 (m, 2H), 7.31-7.35 (m, 2H) ppm; **^{13}C NMR** (75 MHz, $CDCl_3$) δ 13.4, 23.4, 23.9, 53.9, 55.3, 60.9, 63.6, 67.2, 80.3, 87.1, 115.62 (d, *J* = 21.6 Hz, 2 x CH), 116.9, 129.25 (d, *J* = 3.5 Hz), 131.08 (d, *J* = 8.3 Hz, 2 x CH), 162.77 (d, *J* = 248.6 Hz), 164.5, 167.4 ppm; **IR** (neat): υ 3437, 1730, 1681, 1514, 1340, 1242, 1226, 1197, 1108, 1097, 939, 856, 807 cm^{-1}; **HRMS (EI)** m/z: calculated for $C_{19}H_{22}FN_2O_5$ $[M+H]^+$: 377.1513, found: 377.1529.

Synthesis of ethyl 8-cyano-6-hydroxy-3,3-dimethyl-7-(2-nitrophenyl)-5-oxohexahydro-5*H*-oxazolo[3,2-*a*]pyridine-8-carboxylate 12f and 3,3-dimethyl-10-(2-nitrophenyl)-5,8-

dioxotetrahydro-8*H*-6,9-methanooxazolo[3,2-*d*][1,4]oxazepine-9(9a*H*)-carbonitrile 13f: *N*-(1-Hydroxy-2-methylpropan-2-yl)-3-(2-nitrophenyl)oxirane-2-carboxamide **9b** (70 mg, 0.25 mmol), (*E*)-ethyl 2-cyano-3-ethoxyacrylate **10c** (45 mg, 0.375 mmol, 1.5 eq) were dissolved in freshly distilled THF (2 mL). Sodium hydride (3 mg, 0.125 mmol, 0.5 eq.) was then added. The resulting mixture was stirred for 12 hours and was then quenched by addition of a saturated aqueous solution of NH_4Cl (5 mL). The aqueous layer was extracted with EtOAc (3 × 5 mL), the organic layers were combined, brine, dried over $MgSO_4$ and evaporated. The residue was then chromatographed on silica gel to give the desired compound.

The crude material was purified by flash chromatography on silica gel (eluting with Cyclohexane/EtOAc = 70:30) to give the title compound as a yellow solid. Yield: 35%.

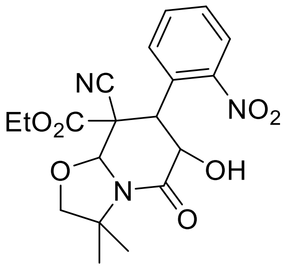

Rf = 0.075 (cyclohexane/EtOAc: 70/30); mp. 210 °C; **^{1}H NMR** (300 MHz, $CDCl_3$) δ 0.88 (t, *J* = 7.2 Hz, 3H), 1.63 (s, 3H), 1.77 (s, 3H), 3.63-3.73 (m, 1H), 3.83-3.91 (m, 1H), 3.89 (bs, 1H), 3.95 (d, *J* = 8.6 Hz, 1H), 4.30 (d, *J* = 8.6 Hz, 1H), 4.54 (d, *J* = 11.1 Hz, 1H), 4.61 (d, *J* = 11.2 Hz, 1H), 5.85 (s, 1H), 7.47-7.56 (m, 2H), 7.67 (t, *J* = 7.1 Hz, 1H), 7.98 (dd, *J* = 8.1, 1.1 Hz, 1H) ppm; **^{13}C NMR** (75 MHz, $CDCl_3$) δ 13.3, 23.8, 23.9, 46.8, 54.6, 61.2, 63.6, 68.3, 80.5, 87.5, 116.3, 125.0, 129.2, 129.4, 129.9, 132.8, 151.0, 164.1, 166.1 ppm; **IR** (neat): υ 3382, 1741, 1688, 1530, 1352, 1248, 945, 672 cm^{-1}; **HRMS (EI)** m/z: calculated for $C_{19}H_{22}N_3O_7$ $[M+H]^+$: 404.1458, found: 404.1462.

The crude material was purified by flash chromatography on silica gel (eluting with Cyclohexane/EtOAc = 70:30) to give the title compound as a yellow solid. Yield: 45%.

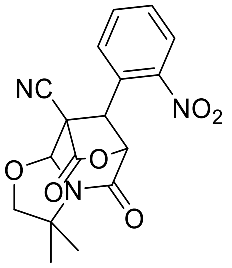

Rf = 0.225 (cyclohexane/EtOAc: 70/30); mp. 225 °C; **^{1}H NMR** (300 MHz, $CDCl_3$) δ 1.11 (s, 3H), 1.31 (s, 3H), 3.86 (d, *J* = 8.7 Hz, 1H), 3.89 (d, *J* = 8.7 Hz, 1H), 4.67 (d, *J* = 8.3 Hz, 1H), 5.59 (s, 1H), 6.70 (d, *J* = 8.3 Hz, 1H), 7.60-7.65 (m, 2H), 7.72 (t, *J* = 7.0 Hz, 1H), 8.37 (d, *J* = 8.3 Hz, 1H) ppm; **^{13}C NMR** (75 MHz, $CDCl_3$) δ 22.9, 25.7, 45.0, 54.3, 60.6, 78.1, 82.2, 92.3, 114.3, 125.7, 126.5, 130.1, 130.2, 134.2, 147.0, 162.9, 165.1 ppm; **IR** (neat): υ 1793, 1705, 1525, 1335, 1339, 1194, 1026, 741 cm^{-1}; **HRMS (EI)** m/z: calculated for $C_{17}H_{16}N_3O_6$ $[M+H]^+$: 358.1039, found: 358.1089.

Synthesis of 3,3,6-trimethyl-5,8-dioxotetrahydro-5*H*,8*H*-furo[3',4':3,4]pyrrolo[2,1-*b*]oxazole-8a(8b*H*)-carbonitrile 14: *N*-(1-Hydroxy-2-methylpropan-2-yl)-3-methyloxirane-2-carboxamide **9d** (45 mg, 0.25 mmol) and (*E*)-ethyl 2-cyano-3-ethoxyacrylate **10c** (44 mg, 0.26 mmol, 1.1 eq) were dissolved in freshly distilled THF (2 mL). Sodium hydride (3 mg, 0.125 mmol, 0.5 eq.) was then added. The mixture was stirred for 12 hours and was then quenched carefully at 0 °C by addition of a saturated aqueous solution of NH_4Cl (2 mL). The aqueous layer was extracted with EtOAc (3 × 5 mL), the organic layers were combined, brine, dried over $MgSO_4$ and solvent was removed under vacuum. The residue was then chromatographed on silica gel to provide the desired compound

The crude material was purified by flash chromatography on silica gel (eluting with Cyclohexane/EtOAc = 70:30) to give the title compound as a white solid. Yield: 53%.

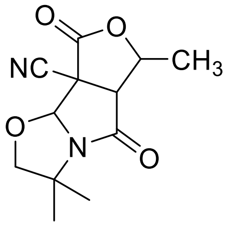

Rf = 0.275 (cyclohexane/EtOAc: 70/30); mp. 230 °C; **^{1}H NMR** (300 MHz, $CDCl_3$) δ 1.44 (s, 3H), 1.59 (s, 3H), 1.63 (d, *J* = 6.7 Hz, 3H), 3.80 (d, *J* = 7.5 Hz, 1H), 3.92 (d, *J* = 8.7 Hz, 1H), 4.00 (d, *J* = 8.7 Hz, 1H), 4.92-5.01 (m, 1H), 5.61 (s, 1H) ppm; **^{13}C NMR** (75 MHz, $CDCl_3$) δ 16.6, 23.1, 25.4, 45.4, 55.0, 60.4, 77.1, 82.5, 92.3, 114.4, 163.3, 165.1 ppm; **IR** (neat): υ 1771, 1711, 1302, 1197, 1045, 1024, 934, 811, 750 cm^{-1}; **HRMS (EI)** m/z: calculated for $C_{12}H_{15}N_2O_4Na$ $[M+Na]^+$: 273.0851, found: 273.0859.

Synthesis of 8-benzoyl-3,3-dimethyl-5-oxo-7-phenylhexahydro-5*H*-oxazolo[3,2-*a*]pyridin-6-yl benzoate 17a: *N*-(1-hydroxy-2-methylpropan-2-yl)-3-phenyloxirane-2-carboxamide **9a** (59 mg, 0.25mmol), 2-(ethoxymethylene)-1,3-diphenylpropane-1,3-*dione* **10d** (70 mg, 0.3 mmol, 1.2 eq) and cesium carbonate (81 mg, 1 eq) were dissolved in freshly distilled CH_3CN (2 mL). The resulting mixture was stirred at 60 °C for 12 hours and was then quenched by addition of a saturated aqueous solution of NH_4Cl (5 mL). The aqueous layer was extracted with EtOAc (3 × 5 mL), the organic layers were combined, brine, dried over $MgSO_4$ and evaporated. The residue was then chromatographed on silica gel to give the desired compound.

The crude material was purified by flash chromatography on silica gel (eluting with Cyclohexane/EtOAc = 70:30) to give the title compound as a white solid. Yield: 85%.

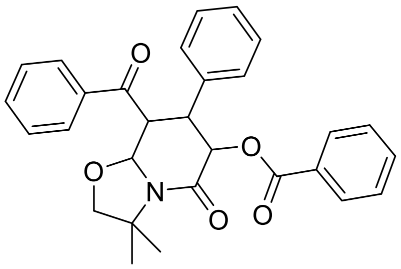

Rf = 0.375 (cyclohexane/EtOAc: 70/30); mp. 248 °C; **^{1}H NMR** (300 MHz, $CDCl_3$) δ 1.52 (s, 3H), 1.56 (s, 3H), 3.58 (d, *J* = 8.9 Hz, 1H), 3.84 (t, *J* = 10.9 Hz, 2H), 4.23 (dd, *J* = 11.9, 8.7 Hz, 1H), 5.20 (d, *J* = 8.6 Hz, 1H), 5.76 (d, *J* = 10.9 Hz, 1H), 6.94-7.03 (m, 3H), 7.09-7.12 (m, 2H), 7.25-7.31 (m, 4H), 7.39-7.45 (m, 2H), 7.68 (dd, *J* = 7.2, 1.3 Hz, 2H), 7.87 (d, *J* = 7.2, 1.3 Hz, 2H) ppm; **^{13}C NMR** (75 MHz, $CDCl_3$) δ 24.0, 24.5, 45.9, 51.4, 60.3, 74.0, 79.8, 89.6, 127.7, 127.9 (2 x CH), 128.2 (2 x CH), 128.3 (2 x CH), 128.5 (2 x CH), 128.7 (2 x CH), 129.4, 129.9 (2 x CH), 133.1, 133.5, 136.5, 137.5, 163.0, 165.6, 197.7 ppm; **IR** (neat): υ 1724, 1664, 1438, 1261, 1222, 1117, 1065 699 cm^{-1}; **HRMS (EI)** m/z: calculated for $C_{29}H_{28}NO_5$ $[M+H]^+$: 470.1967, found: 470.1975.

Synthesis of 8-benzoyl-7-(4-fluorophenyl)-3,3-dimethyl-5-oxohexahydro-5*H*-oxazolo[3,2-*a*]pyridin-6-yl benzoate 17b: 3-(4-fluorophenyl)-*N*-(1-hydroxy-2-methylpropan-2-yl)oxirane-2-carboxamide **9c** (63 mg, 0.25mmol), 2-(ethoxymethylene)-1,3-diphenylpropane-1,3-dione **10d** (70 mg, 0.275 mmol, 1.1 eq) and cesium carbonate (81 mg, 1 eq) were dissolved in freshly distilled CH_3CN (2 mL). The resulting mixture was stirred at 60 °C for 12 hours and was then quenched by addition of a saturated aqueous solution of NH_4Cl (5 mL). The aqueous layer was extracted with EtOAc (3 × 5 mL), the organic layers were combined, brine, dried over $MgSO_4$ and evaporated. The residue was then chromatographed on silica gel to give the desired compound.

The crude material was purified by flash chromatography on silica gel (eluting with Cyclohexane/EtOAc = 70:30) to give the title compound as a white solid. Yield: 80%.

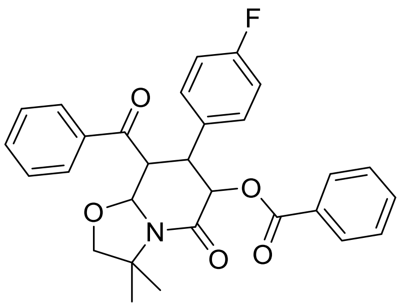

Rf = 0.35 (cyclohexane/EtOAc: 70/30); mp. 267 °C; **^{1}H NMR** (300 MHz, $CDCl_3$) δ 1.61 (s, 3H), 1.65 (s, 3H), 3.68 (d, *J* = 8.9 Hz, 1H), 3.91 (d, *J* = 11.6 Hz, 1H), 3.96 (d, *J* = 8.7 Hz, 1H), 4.32 (dd, *J* = 11.9, 8.6 Hz, 1H), 5.28 (d, *J* = 8.6 Hz, 1H), 5.86 (d, *J* = 11.0 Hz, 1H), 6.80 (t, *J* = 8.6 Hz, 2H), 7.19 (dd, *J* = 8.6, 5.2 Hz, 2H), 7.37-7.42 (m, 4H), 7.53 (dd, *J* = 15.3, 7.8 Hz, 2H), 7.79 (dd, *J* = 7.3, 1.3 Hz, 2H), 7.97 (d, *J* = 7.3, 1.3 Hz, 2H) ppm; **^{13}C NMR** (75 MHz, $CDCl_3$) δ 24.0, 24.4, 45.2, 51.4, 60.4, 73.8, 79.8, 89.6, 115.7 (d, *J* = 21.6 Hz, 2 x CH), 128.3 (4 x CH), 128.4, 128.6 (2 x CH), 129.5 (d, *J* = 8.2 Hz, 2 x CH), 129.9 (2 x CH), 130.1, 132.3 (d, *J* = 3.3 Hz), 133.2, 133.7, 162.0 (d, *J* = 246.6 Hz), 163.9, 165.6, 197.6 ppm; **IR** (neat): υ 1744, 1664, 1444, 1259, 1222, 704 cm^{-1}; **HRMS (EI)** m/z: calculated for $C_{29}H_{27}FNO_5$ $[M+H]^+$: 488.1873, found: 488.1877.

Synthesis of 8-benzoyl-3,3-dimethyl-5-oxo-7-phenylhexahydro-5*H*-oxazolo[3,2-*a*]pyridin-6-yl ethyl carbonate 18: *N*-(1-hydroxy-2-methylpropan-2-yl)-3-phenyloxirane-2-carboxamide **9a** (59 mg, 0.25mmol), ethyl 2-benzoyl-3-ethoxyacrylate **10e** (68 mg, 0.275 mmol, 1.1 eq) and cesium carbonate (81 mg, 1 eq) were dissolved in freshly distilled CH_3CN (2 mL). The resulting mixture was stirred at 50 °C for 12 hours and was then quenched by addition of a saturated aqueous solution of NH_4Cl (5 mL). The aqueous layer was extracted with EtOAc (3 × 5 mL), the organic layers were combined, brine, dried over $MgSO_4$ and evaporated. The residue was then chromatographed on silica gel to give the desired compound.

The crude material was purified by flash chromatography on silica gel (eluting with Cyclohexane/EtOAc = 70:30) to give the title compound as a white solid. Yield: 59%.

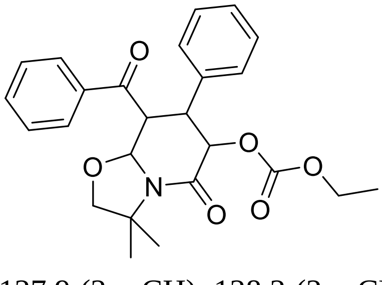

Rf = 0.275 (cyclohexane/EtOAc: 70/30); mp. 220 °C; **^{1}H NMR** (300 MHz, $CDCl_3$) δ 1.23 (t, *J* = 7.1 Hz, 3H), 1.60 (s, 3H), 1.65 (s, 3H), 3.64 (d, *J* = 8.9 Hz, 1H), 3.76 (t, *J* = 11.5 Hz, 1H), 3.94 (d, *J* = 8.8 Hz, 1H), 4.05-4.24 (m, 3H), 5.18 (d, *J* = 8.6 Hz, 1H), 5.47 (d, *J* = 11.0 Hz, 1H), 7.09-7.21 (m, 5H), 7.37 (t, *J* = 7.6 Hz, 2H), 7.51 (t, *J* = 7.4 Hz, 1H), 7.74 (dd, *J* = 7.2, 1.3 Hz, 2H) ppm; **^{13}C NMR** (75 MHz, $CDCl_3$) δ 14.0, 23.9, 24.5, 45.6, 51.3, 60.3, 64.6, 76.3, 79.8, 89.5, 127.8, 127.9 (2 x CH), 128.2 (2 x CH), 128.5 (2 x CH), 128.7 (2 x CH), 133.5, 136.2, 137.5, 154.6, 162.7, 197.4 ppm; **IR** (neat): υ 1745, 1663, 1444, 1258, 1221, 1039, 764 cm^{-1}; **HRMS (EI)** m/z: calculated for $C_{25}H_{28}NO_6$ $[M+H]^+$: 438.1917, found: 438.1911.

4. X-ray Crystal Structure Determination of compounds 13a, 12e, 14 and 18:

Crystals were selected under polarizing optical microscope and mounted on MicroMount needles (MiTiGen) for single-crystal X-ray diffraction experiments. X-ray intensity data were collected on a Bruker APEX II Quazar diffractometer (4 circle Kappa goniometer, CCD detector) using Iμs microfocus source (Mo-K_α radiation with λ = 0.71073 Å) at 296 K. The structure solutions were obtained by direct methods, developed by successive difference Fourier syntheses, and refined by full-matrix least-squares on all F^2 data using SHELX program suite[1] in Bruker APEX2 interface. Details of the structure determinations are given in Table 1.

Crystallographic data (excluding structure factors) for all structures have been deposited at the Cambridge Crystallographic Data Centre, CCDC 2333920 ($C_{12}H_{14}N_2O$ (**14**)), 2333921 ($C_{19}H_{21}NO_6$ (**13a**)), 2333922 ($C_{19}H_{21}FN_2O_5$ (**12e**)) and 2333923 ($C_{25}H_{27}NO_6$ (**18**)). Copies of the data can be obtained, free of charge, on application to CCDC, 12 Union Road, Cambridge CB2 1EZ, UK, (fax: +44 1223 336033 or e-mail: deposit@ccdc.cam.ac.uk).

Table 1. Crystal data and structure refinement for four compounds

Identification code	$C_{12}H_{14}N_2O$ **14**	$C_{19}H_{21}NO_6$ **13a**	$C_{19}H_{21}FN_2O_5$ **12e**	$C_{25}H_{27}NO_6$ **18**
CCDC number	2333920	2333921	2333922	2333923
Formula mass (g.mol^{-1})	250.25	359.37	376.38	437.48
Unit cell dimensions				
a (Å) =	11.9657(17)	10.1811(5)	8.8874(7)	19.706(3)
b (Å) =	9.7644(12)	15.0709(6)	11.0915(8	5.9447(8)
c (Å) =	11.0251(15)	11.8115(5)	18.9766(13)	21.801(3)
β (°) =	111.305(4)	100.105(2)	97.496(2)	116.513(4)
Volume, Z	1200.1(3), 4	1784.2(2), 4	1854.6(2), 4	2285.4(5), 4
Calculated density (g.cm^{-3})	1.385	1.338	1.348	1.271
Crystal system, Space group	Monoclinic, $P2_1/c$			Monoclinic, $P2_1/n$
Temperature (K), scan method	296, 2θ/ω			
2θ range for data collection (°)	5.6-51.4	4.4-55.0	4.3-50.4	2.7-55.0
$(hkl)_{min}$ $(hkl)_{max}$	(-14 -11 -13) (14 11 13)	(-13 -19 -15) (13 19 15)	(-10 -13 -21) (10 13 22)	(-24 -7 -28) (25, 7, 28)
coefficient (mm^{-1})	0.105	0.100	0.105	0.091
Reflections (I>2σ(I)), refined numbers	1221, 166	3033, 238	1837, 249	3392, 292
Goodness of fit (F^2)	0.980	1.031	0.851	1.029
R_1 [I >2σ(I)], wR_2 (all data)	0.0474, 0.1179	0.0437, 0.1231	0.0441, 0.0981	0.0494, 0.1457

[1] G.M. Sheldrick, " SHELXL-2014 ", Program for crystal structure determination, Göttingen Univ., Germany (2014).

Compound 13a

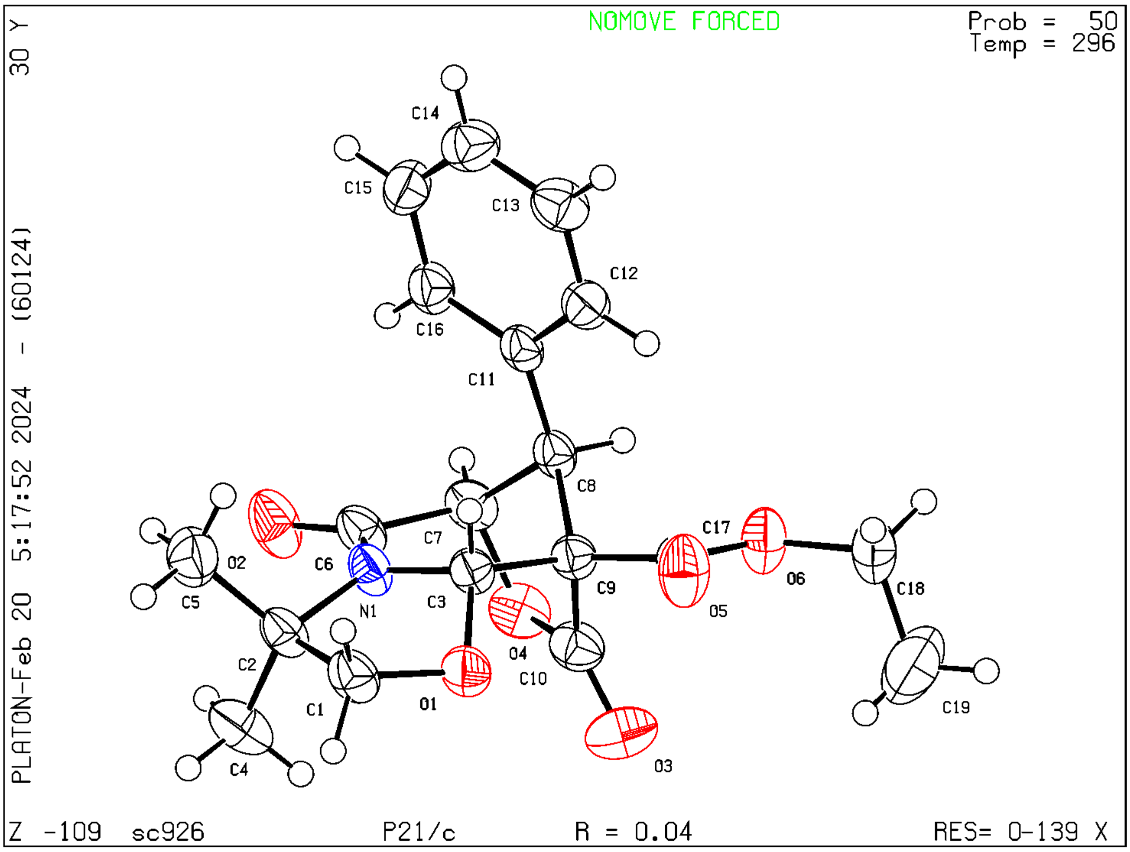

Compound 14

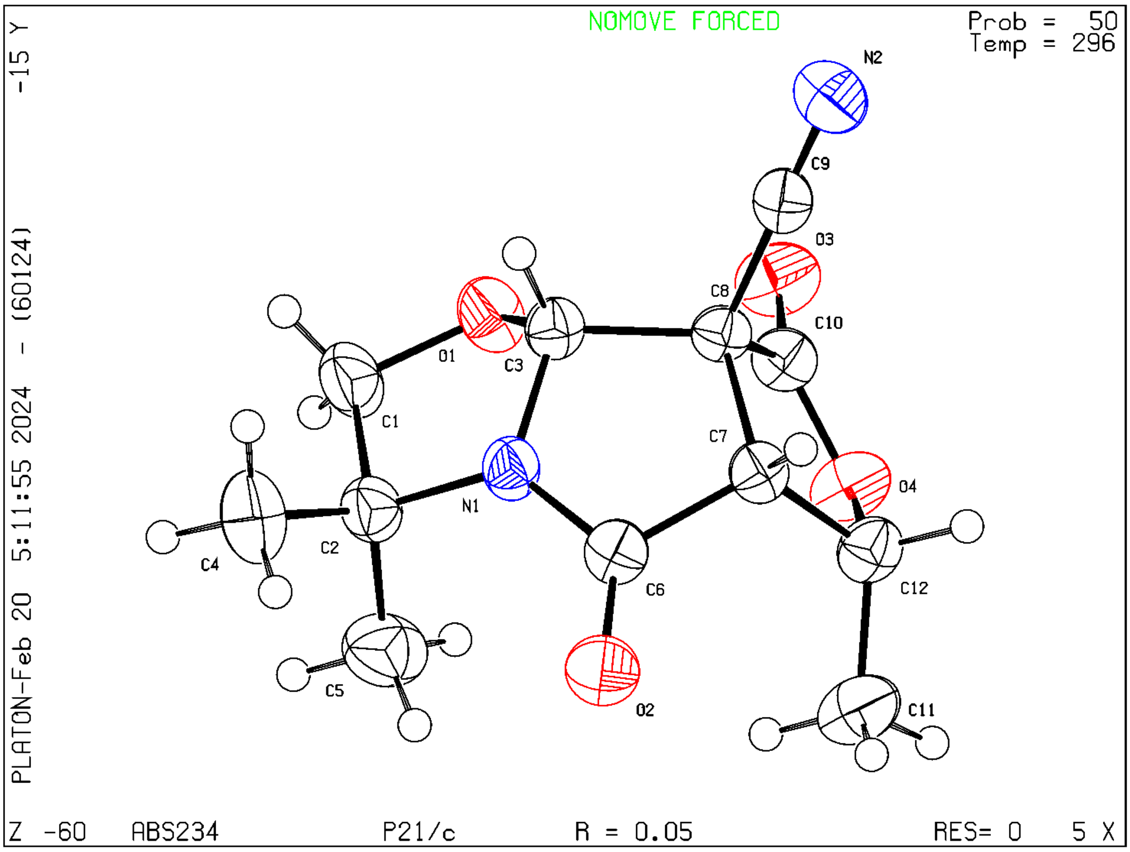
NOMOVE FORCED
Prob = 50
Temp = 296
-15 Y
PLATON-Feb 20 5:11:55 2024 - (60124)
N2
C9
O3
C8
C10
O1
C3
C1
C7
O4
N1
C2
C4
C6
C12
C5
O2
C11
Z -60
ABS234
P21/c
R = 0.05
RES= 0
5 X

Compound 12e

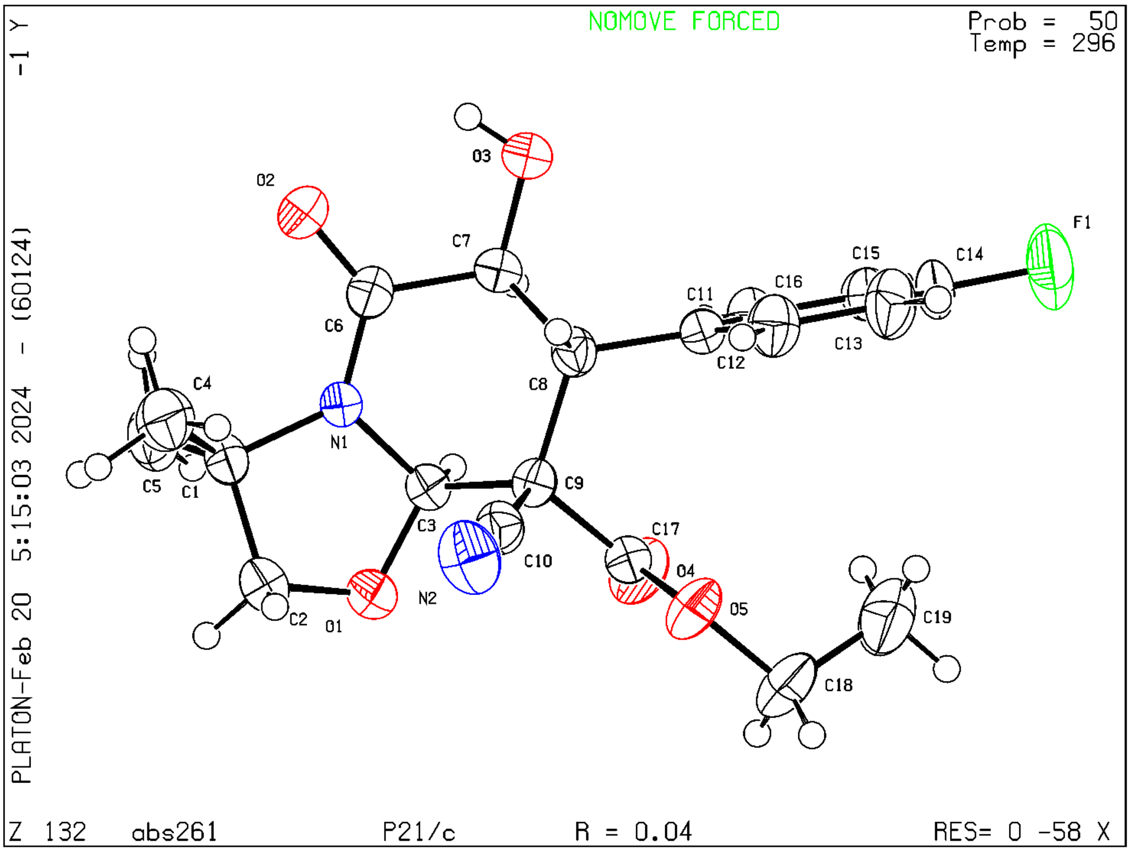

Compound 18

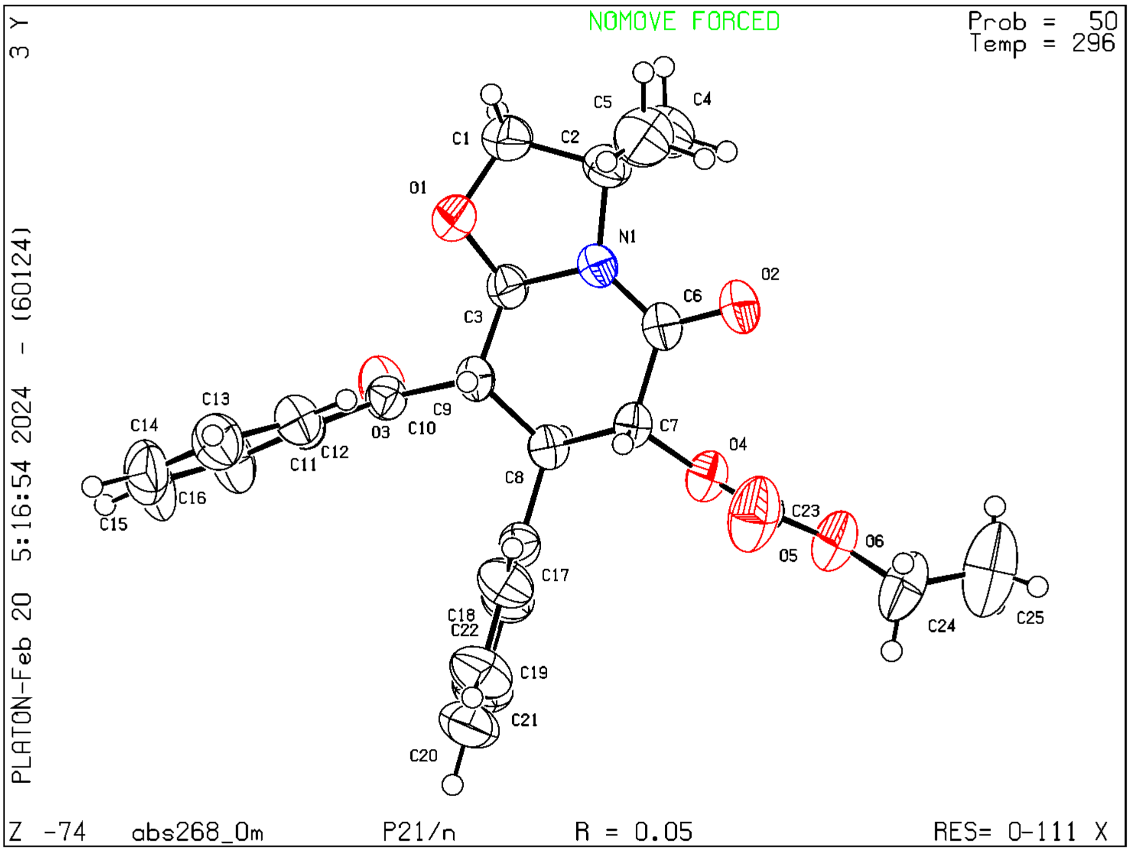
NOMOVE FORCED
Prob = 50
Temp = 296
3 Y
PLATON-Feb 20 5:16:54 2024 - (60124)
C1
C2
C3
C4
C5
C6
C7
C8
C9
C10
C11
C12
C13
C14
C15
C16
C17
C18
C19
C20
C21
C22
C23
C24
C25
N1
O1
O2
O3
O4
O5
O6
Z -74 abs268_0m P21/n R = 0.05 RES= 0-111 X

5. ^{1}H and ^{13}C NMR spectra

^{1}H NMR of Compound 9a (300 MHz, $CDCl_3$)

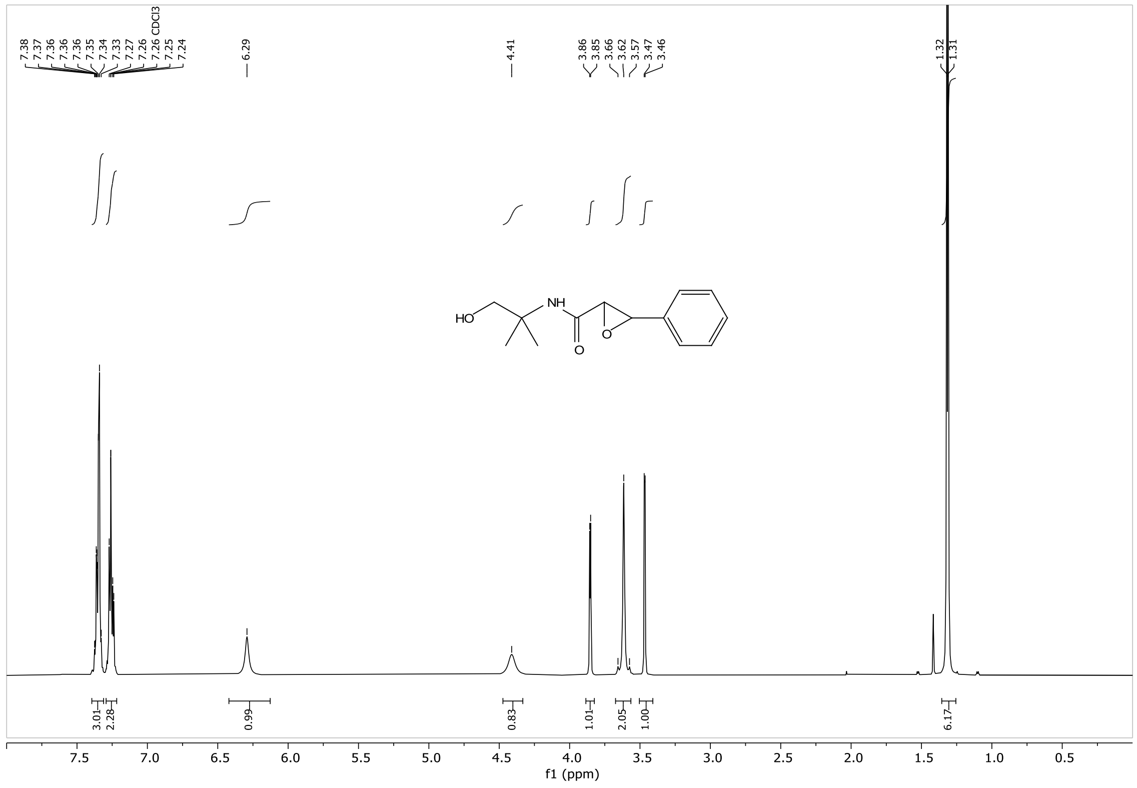

^{13}C NMR of Compound 9a (75 MHz, $CDCl_3$)

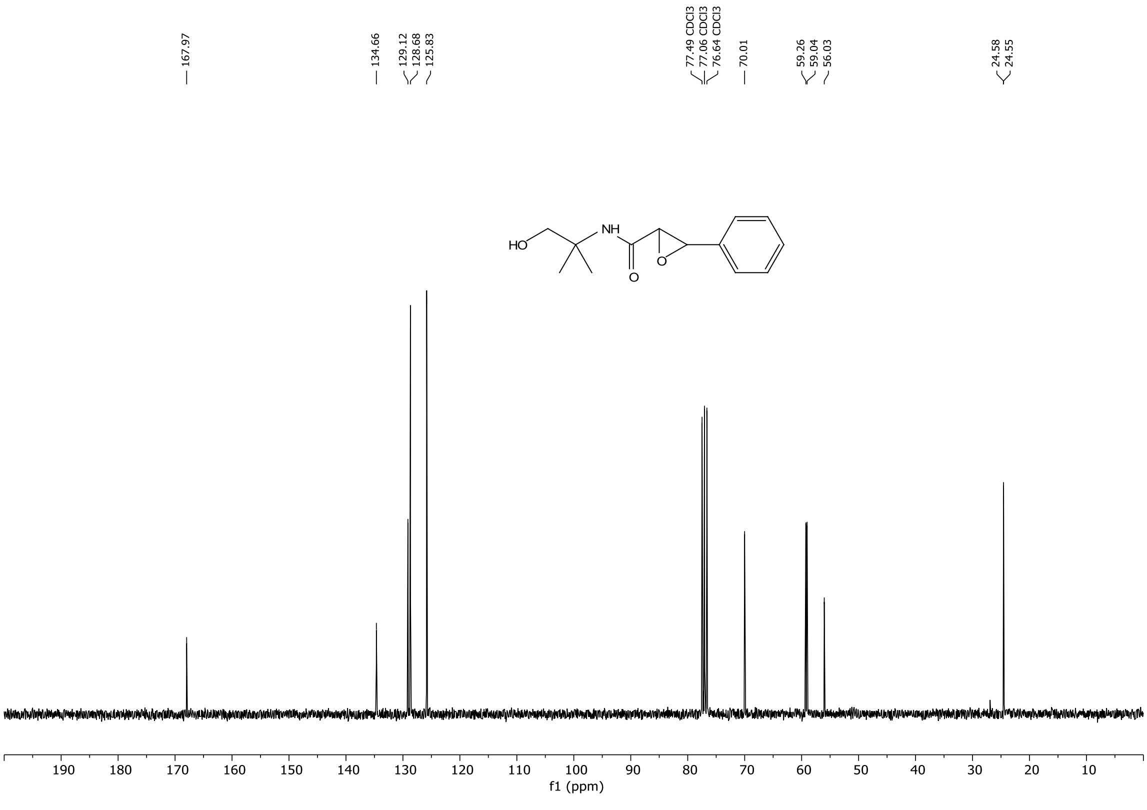

^{1}H NMR of Compound 9b (300 MHz, $CDCl_3$)

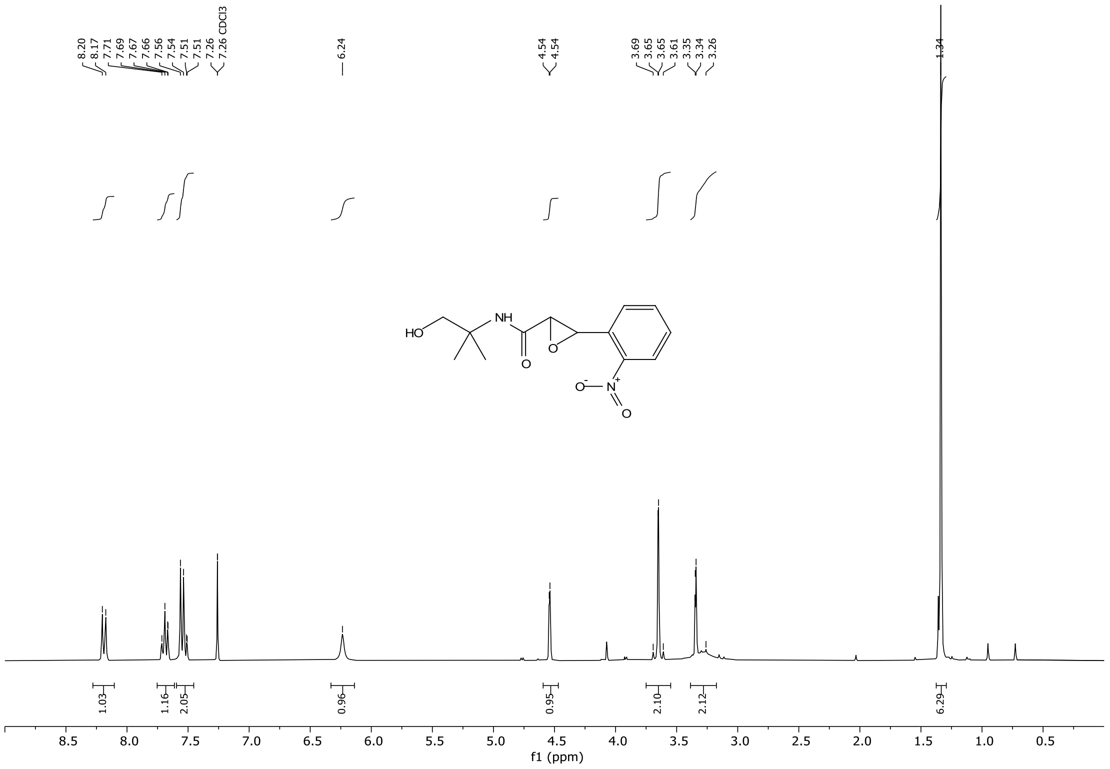

^{13}C NMR of Compound 9b (75 MHz, $CDCl_3$)

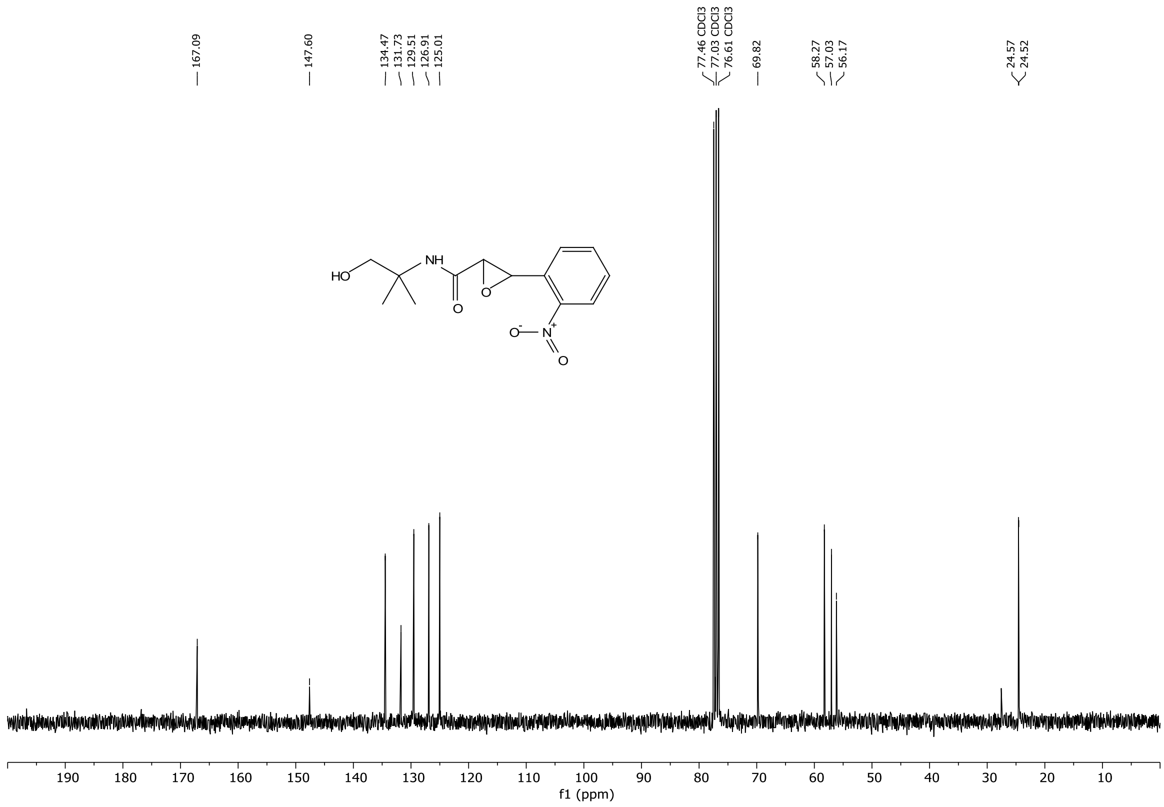

^{1}H NMR of Compound 9c (300 MHz, $CDCl_3$)

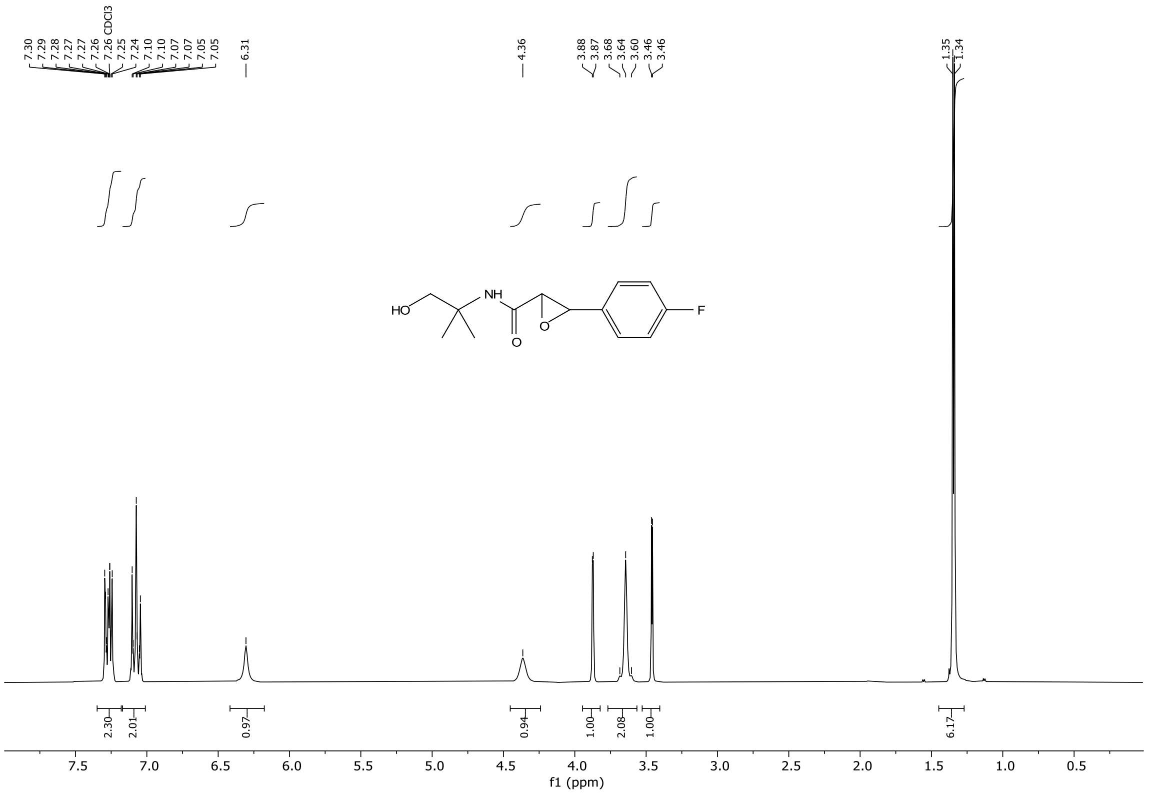

^{13}C NMR of Compound 9c (75 MHz, $CDCl_3$)

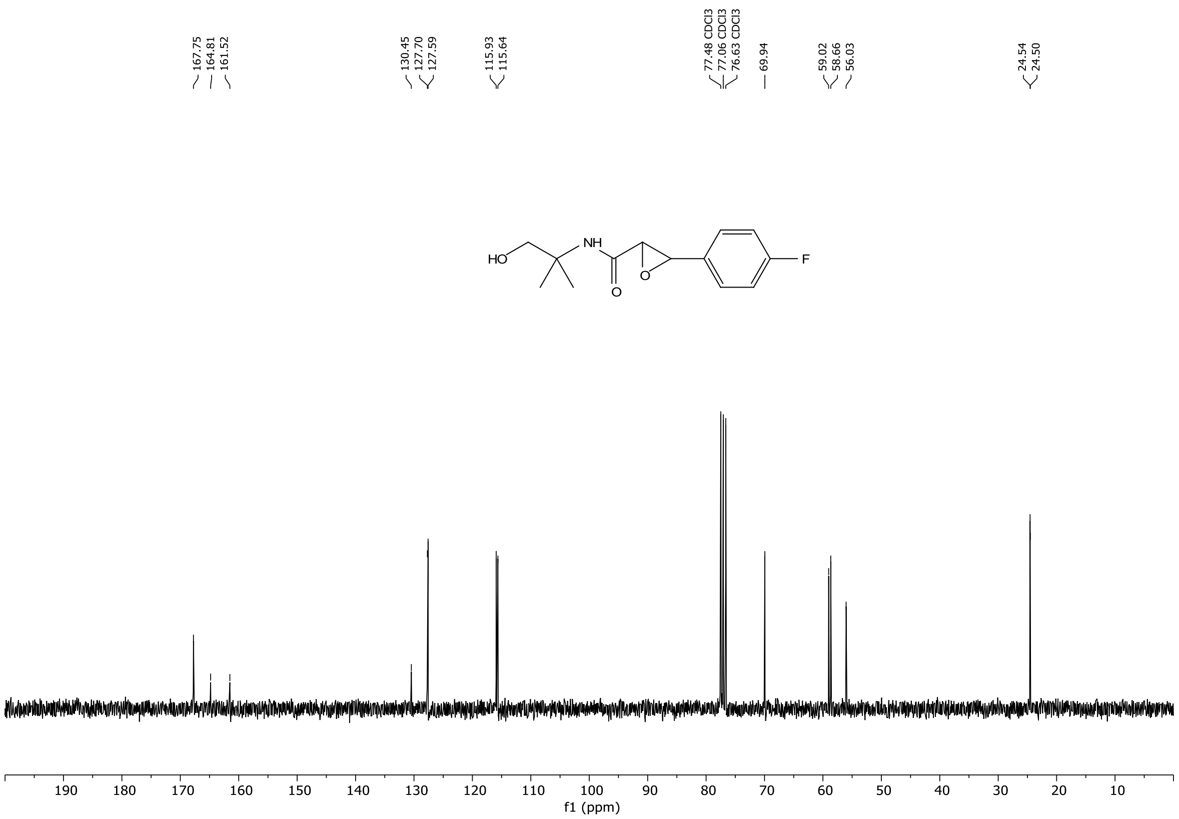

^{1}H NMR of Compound 9d (300 MHz, $CDCl_3$)

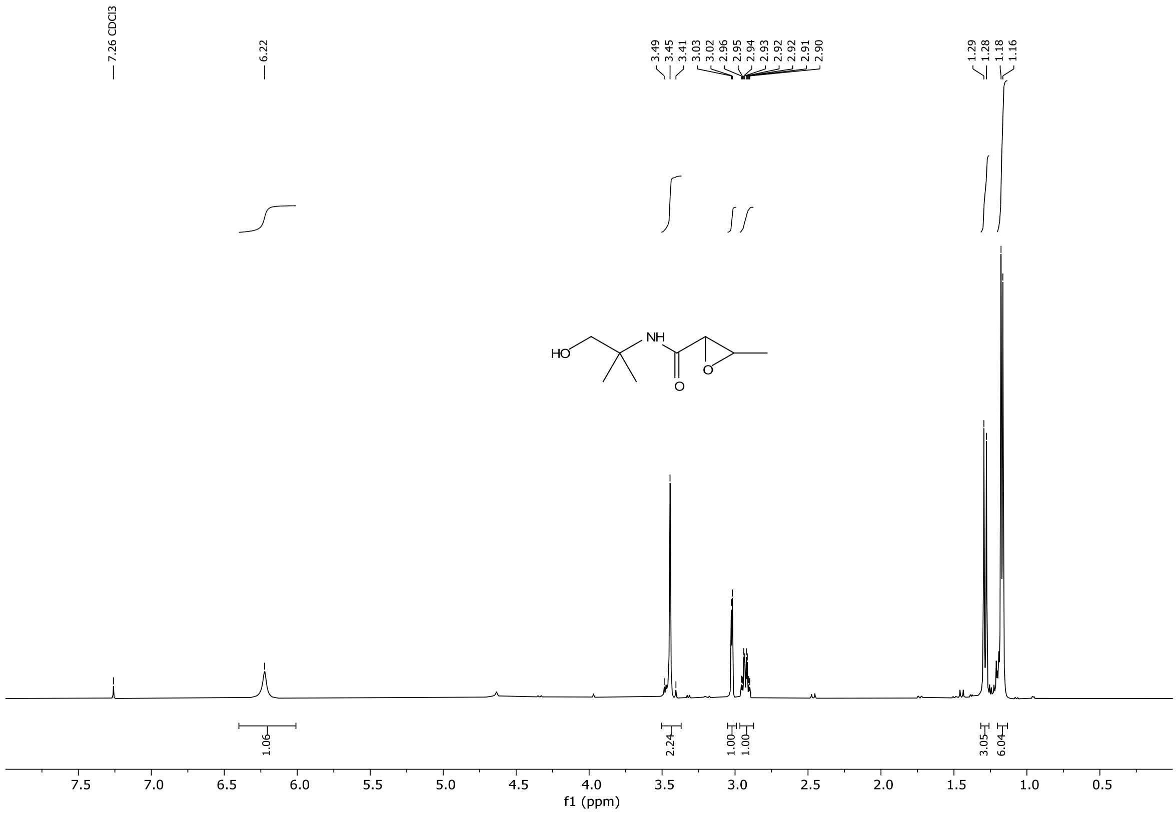

^{13}C NMR of Compound 9d (75 MHz, $CDCl_3$)

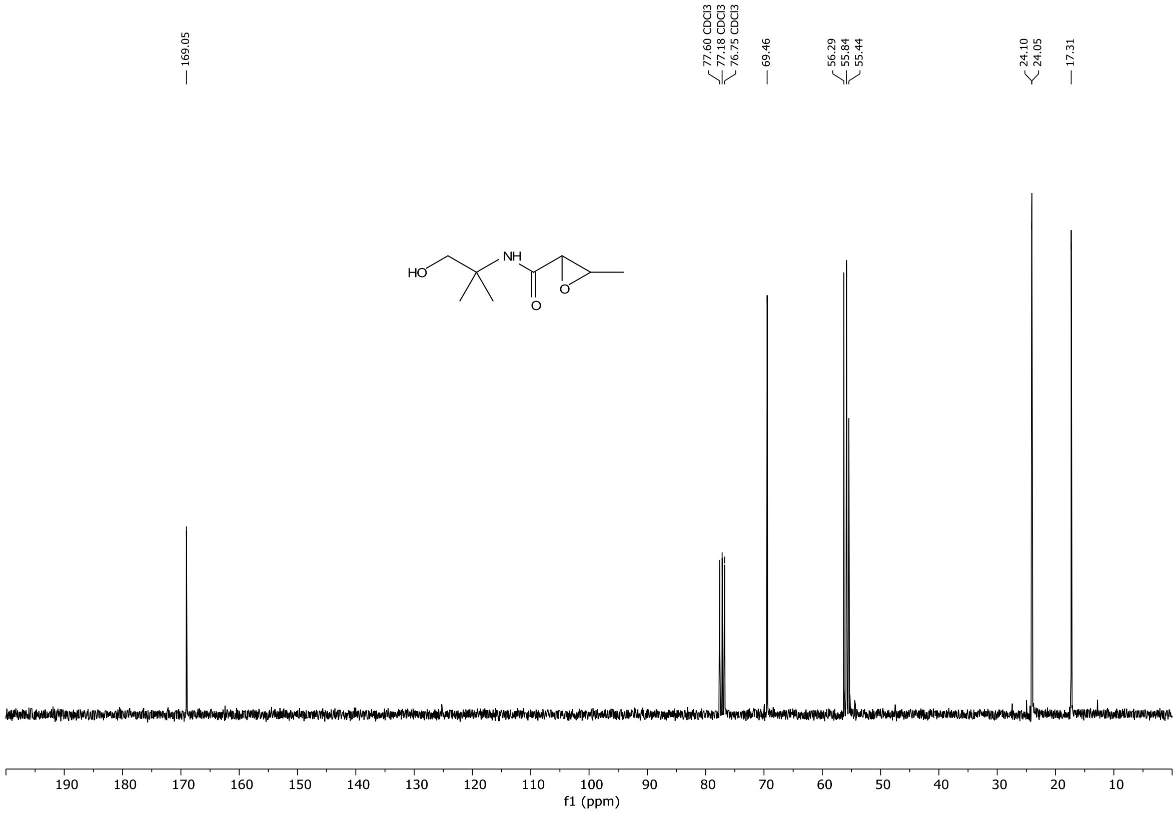

^{1}H NMR of Compound 12a (300 MHz, $CDCl_3$)

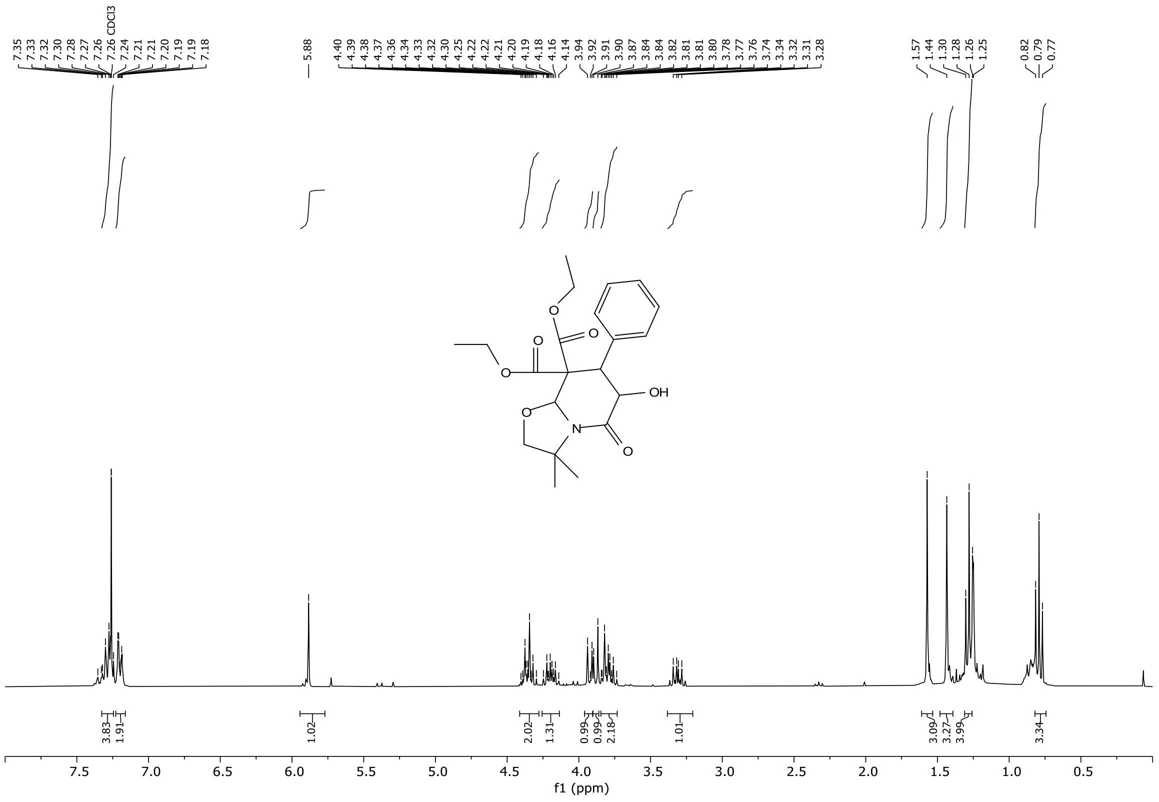

^{13}C NMR of Compound 12a (75 MHz, $CDCl_3$)

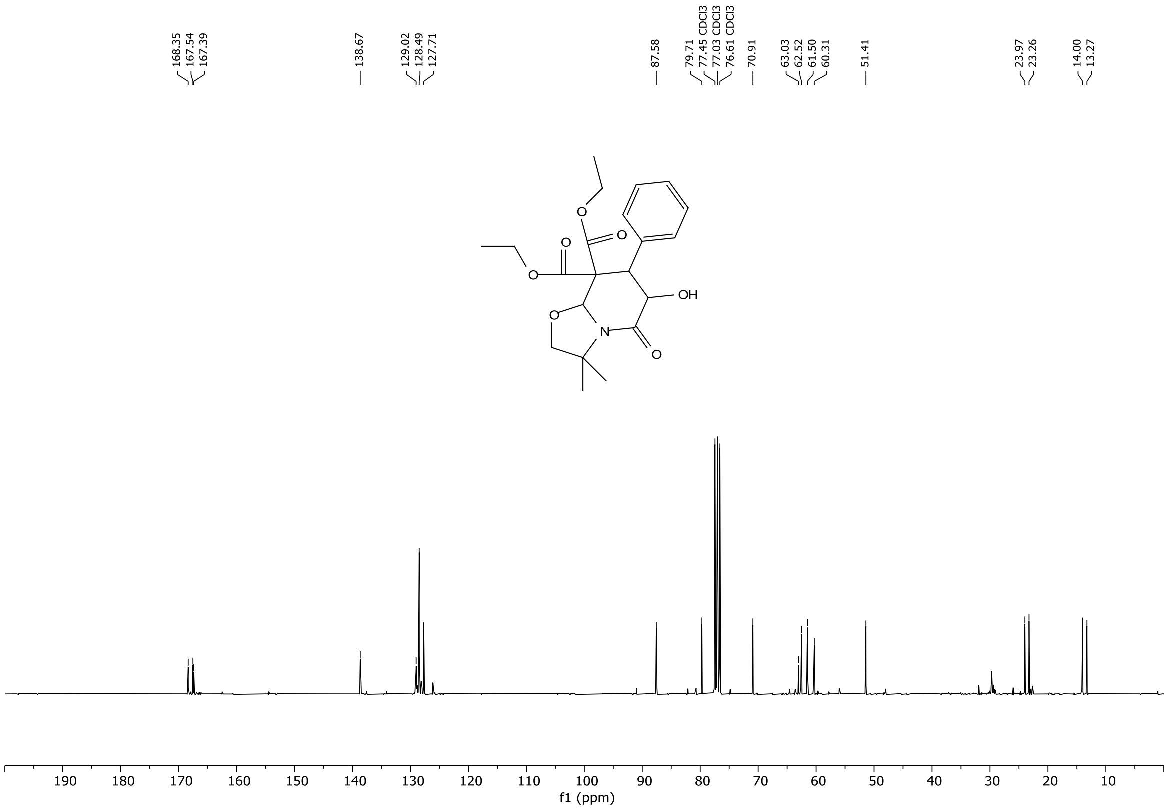

^{1}H NMR of Compound 13a (300 MHz, $CDCl_3$)

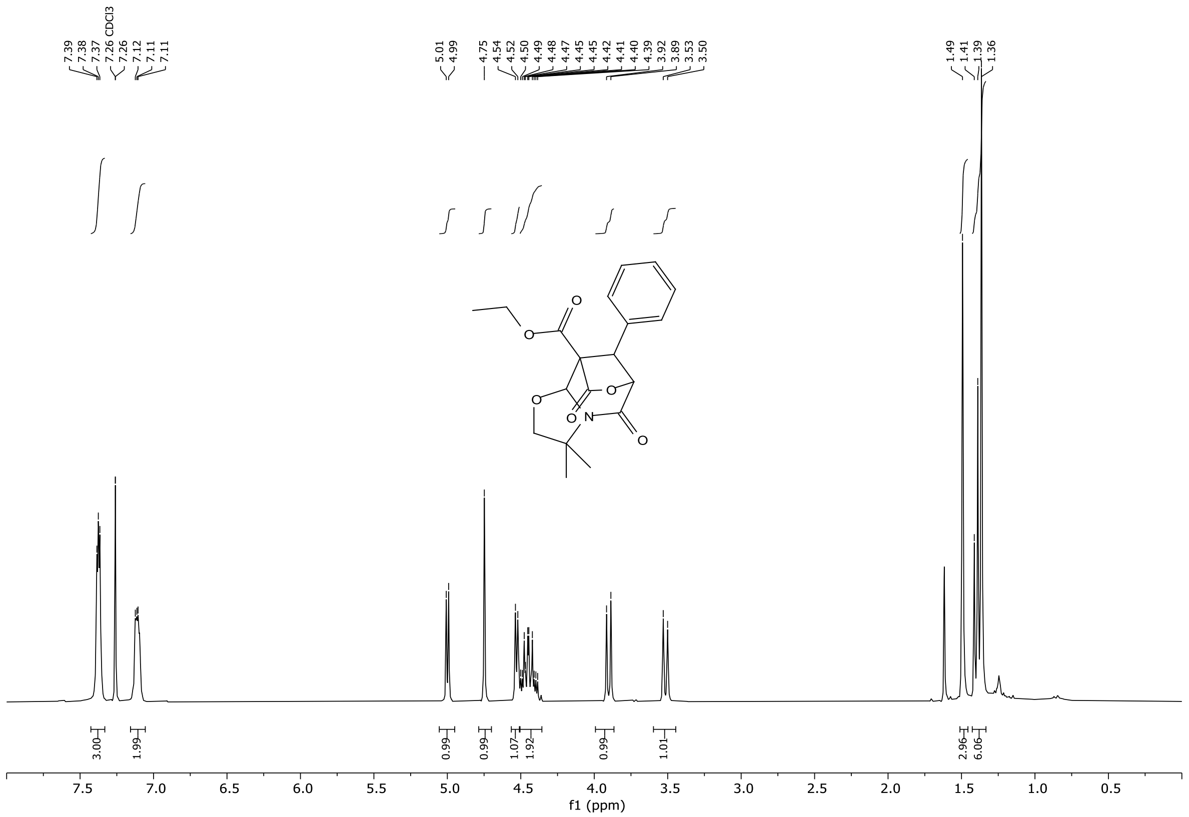

^{13}C NMR of Compound 13a (75 MHz, $CDCl_3$)

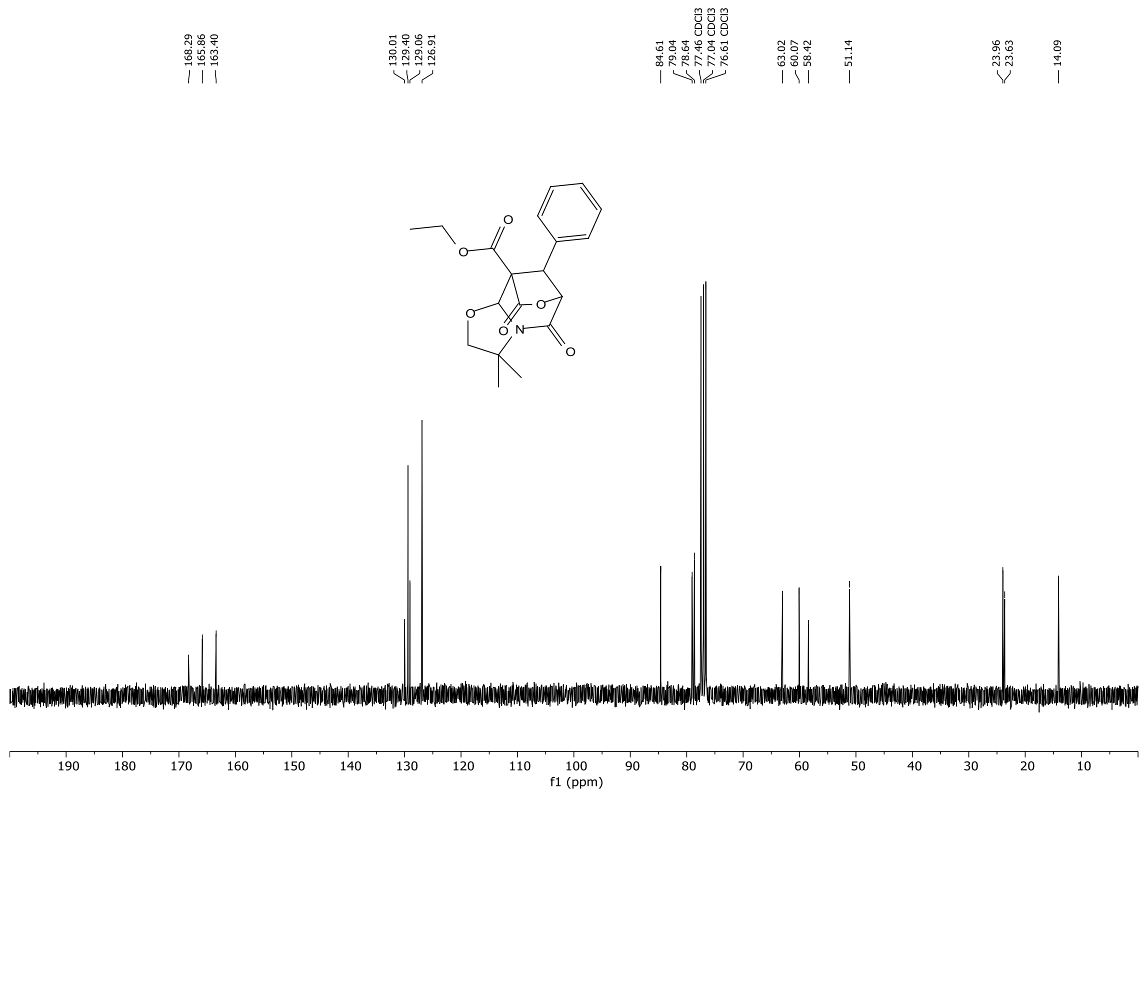

^{1}H NMR of Compound 13b (300 MHz, $CDCl_3$)

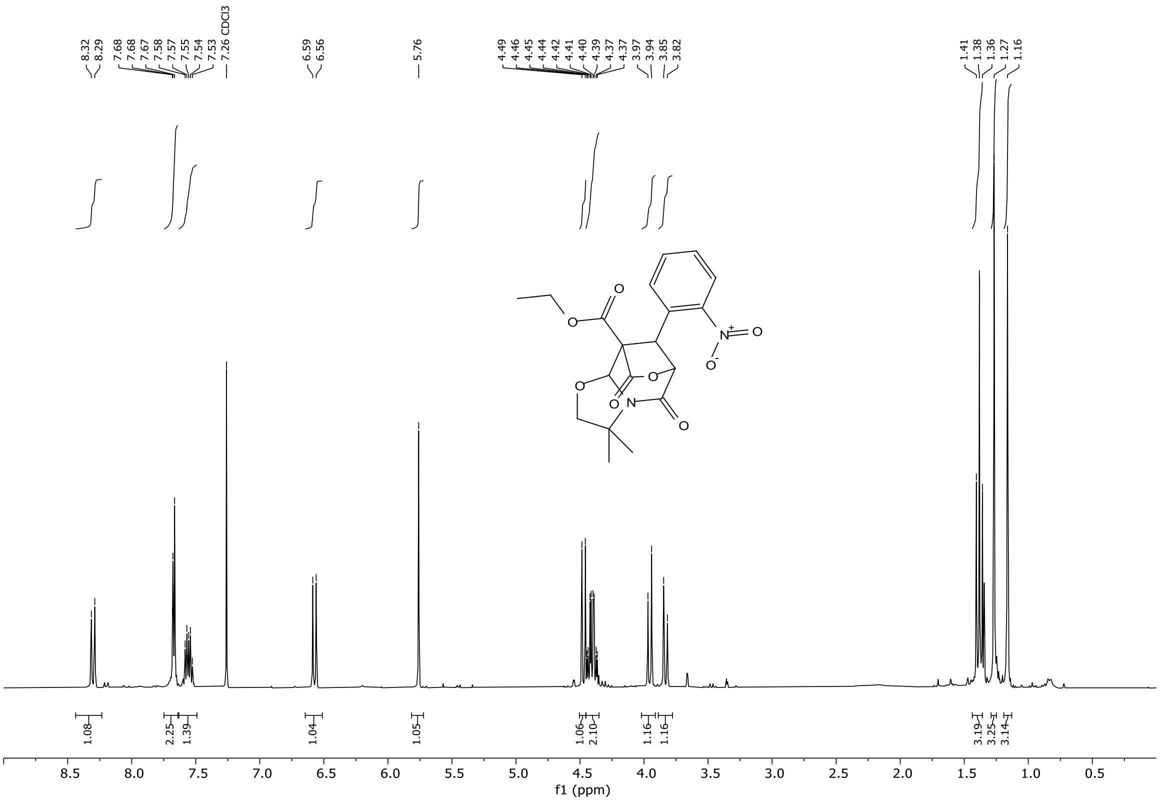

^{13}C NMR of Compound 13b (75 MHz, $CDCl_3$)

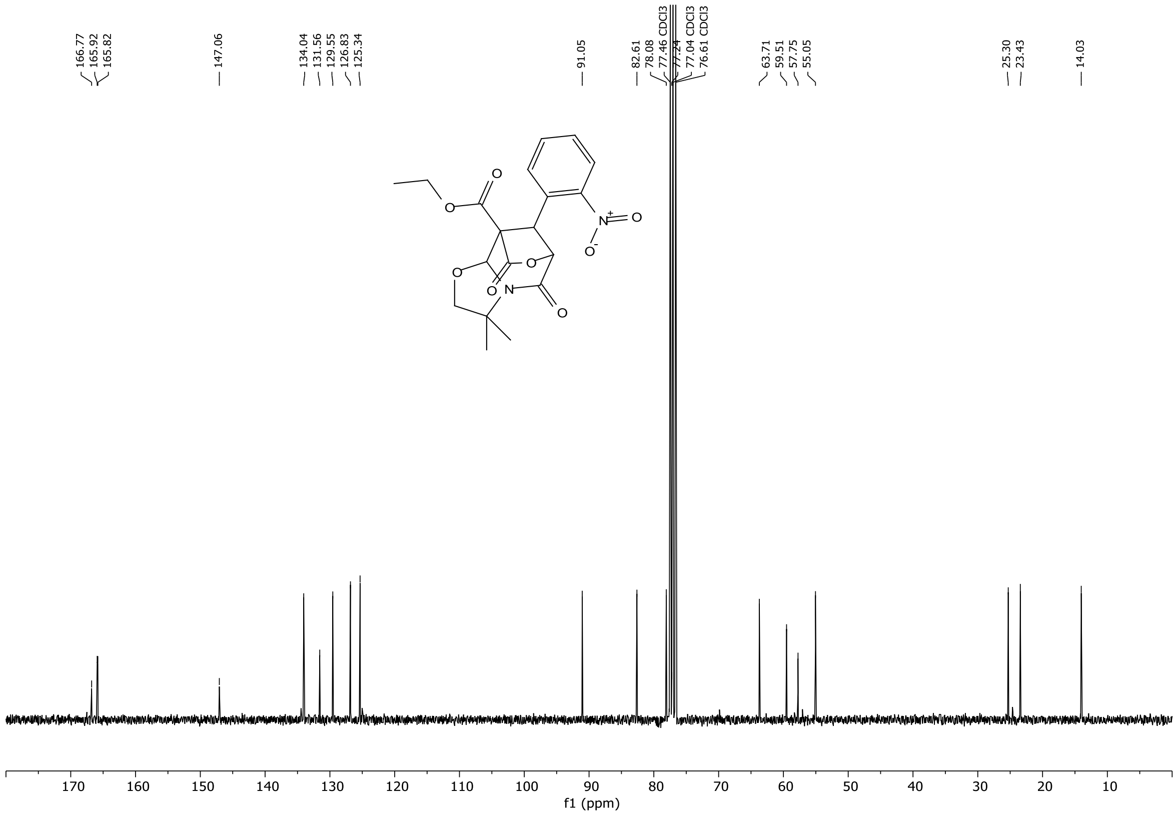

^{1}H NMR of Compound 12c (300 MHz, $CDCl_3$)

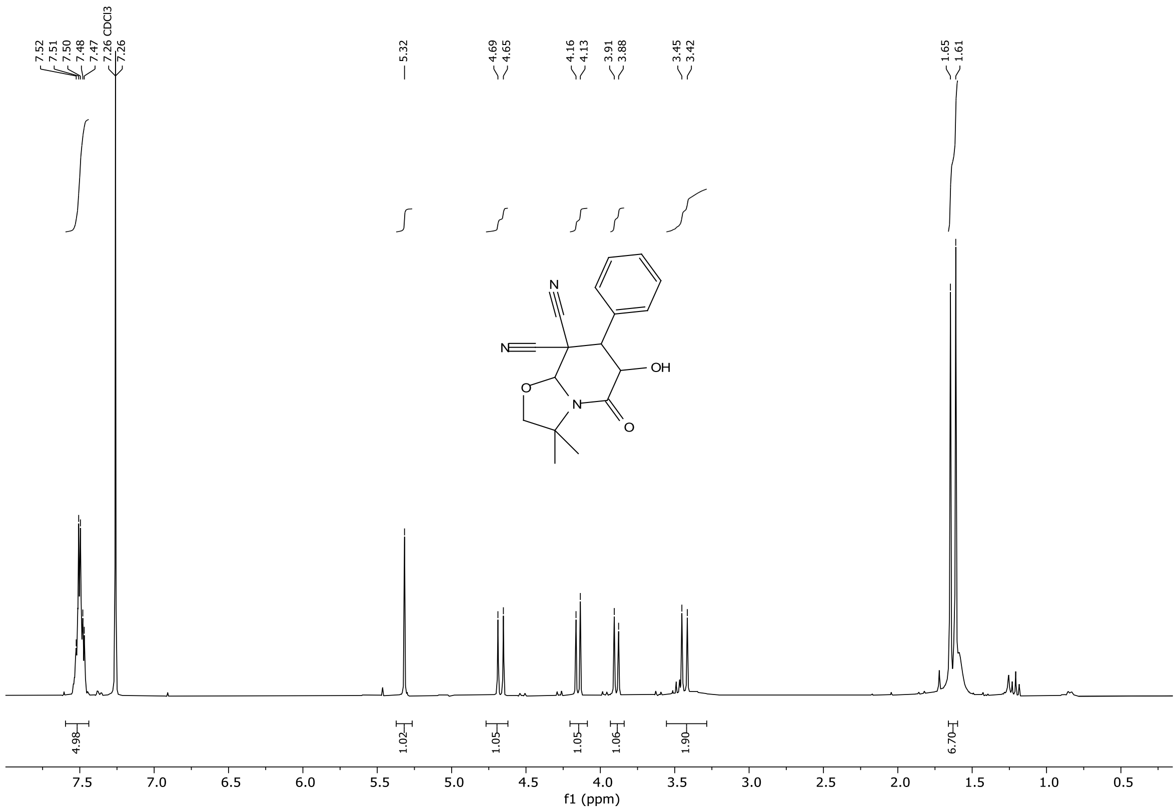

^{13}C NMR of Compound 12c (75 MHz, $CDCl_3$)

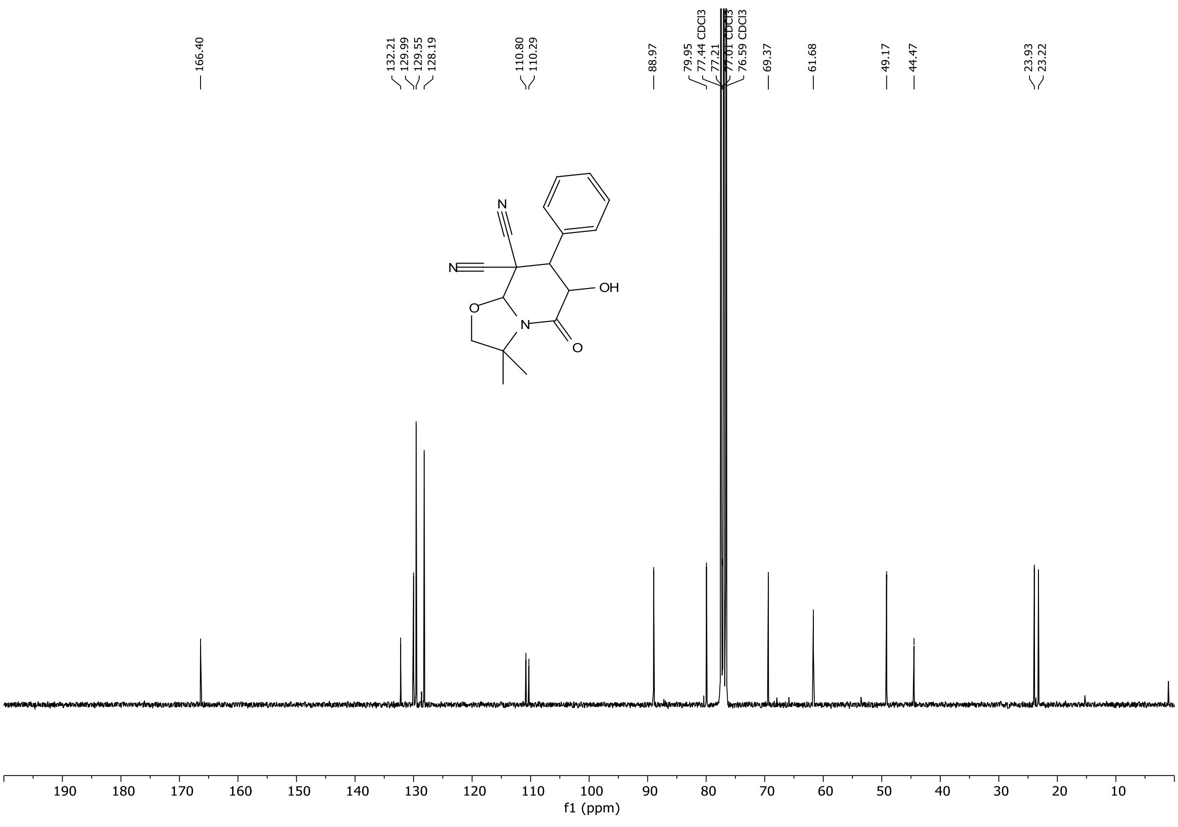

^{1}H NMR of Compound 12d (300 MHz, $CDCl_3$)

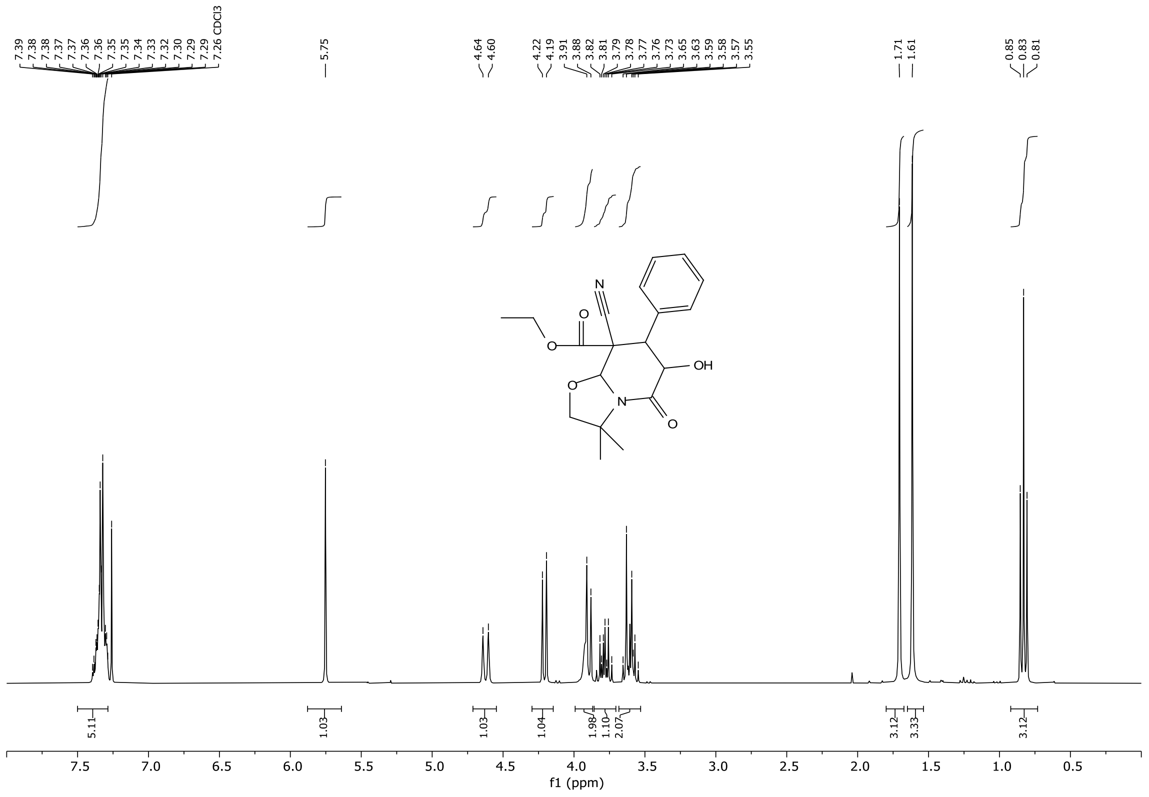

^{13}C NMR of Compound 12b (75 MHz, $CDCl_3$)

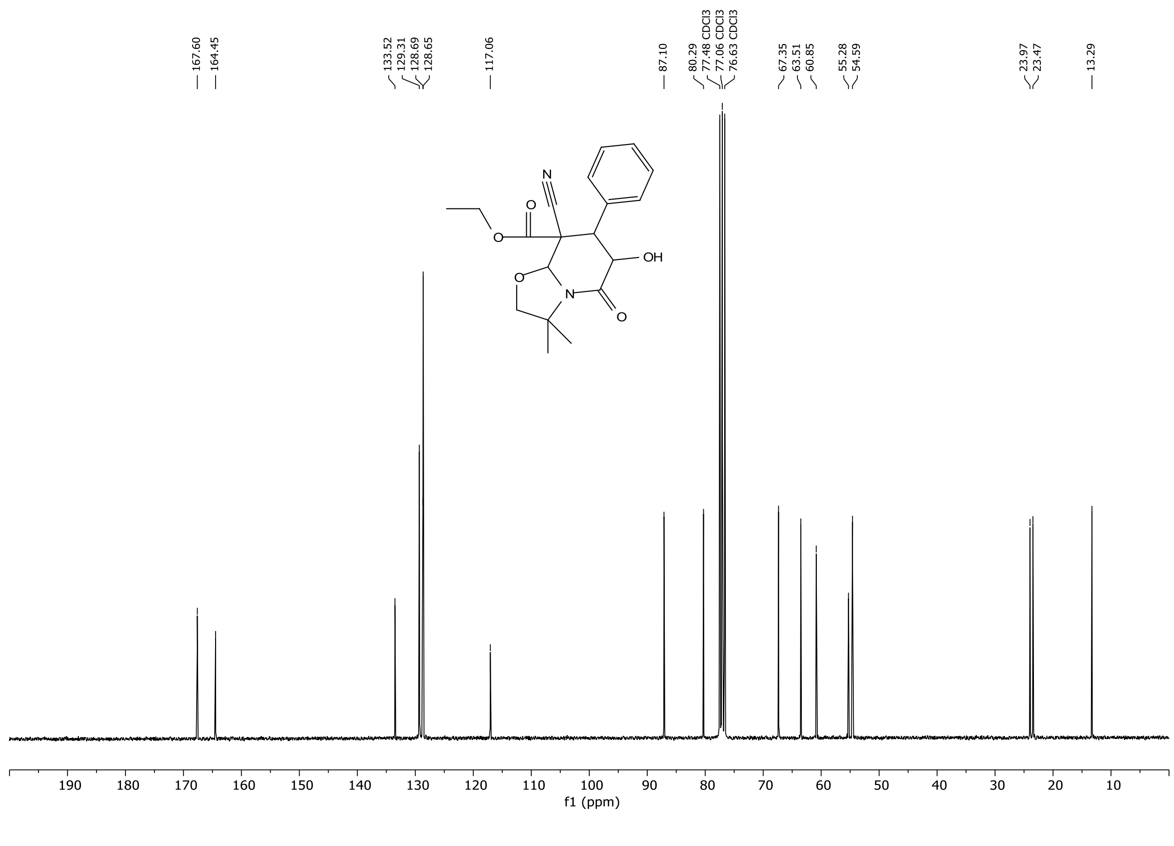

^{1}H NMR of Compound 12e (300 MHz, $CDCl_3$)

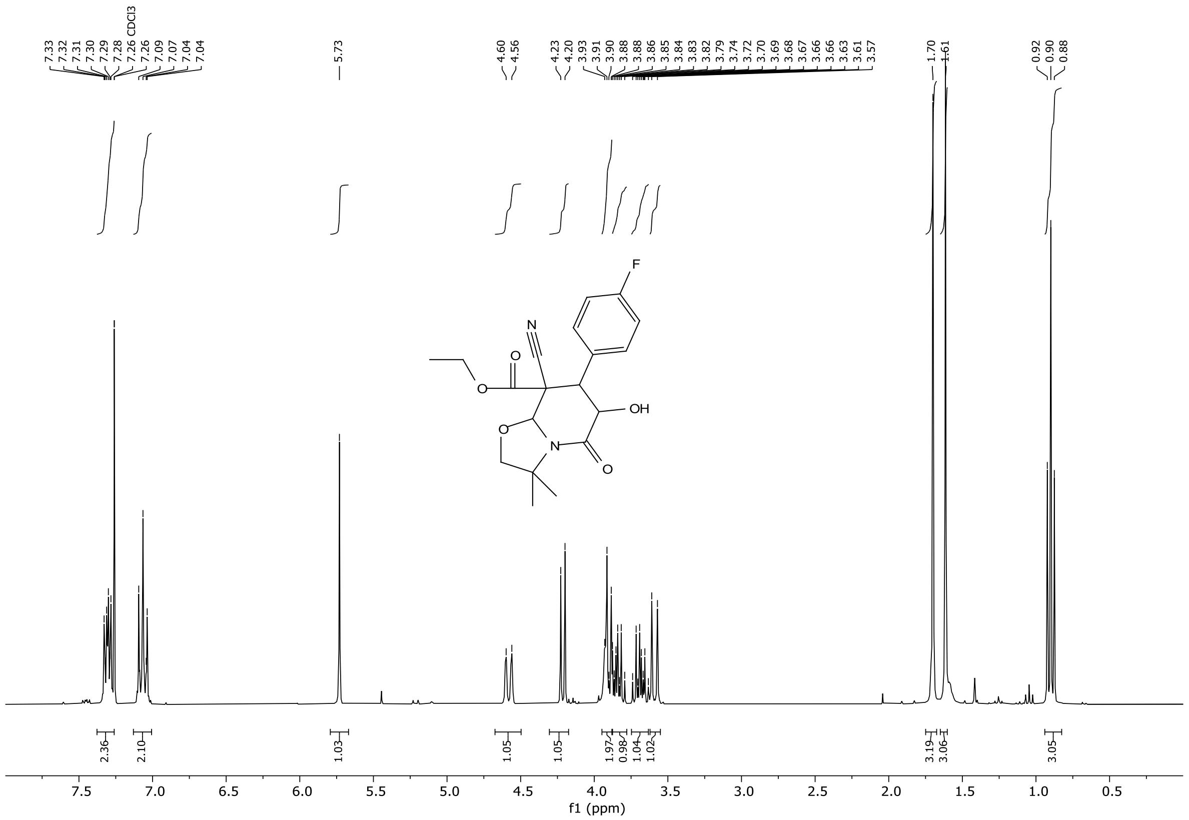

^{13}C NMR of Compound 12e (75 MHz, $CDCl_3$)

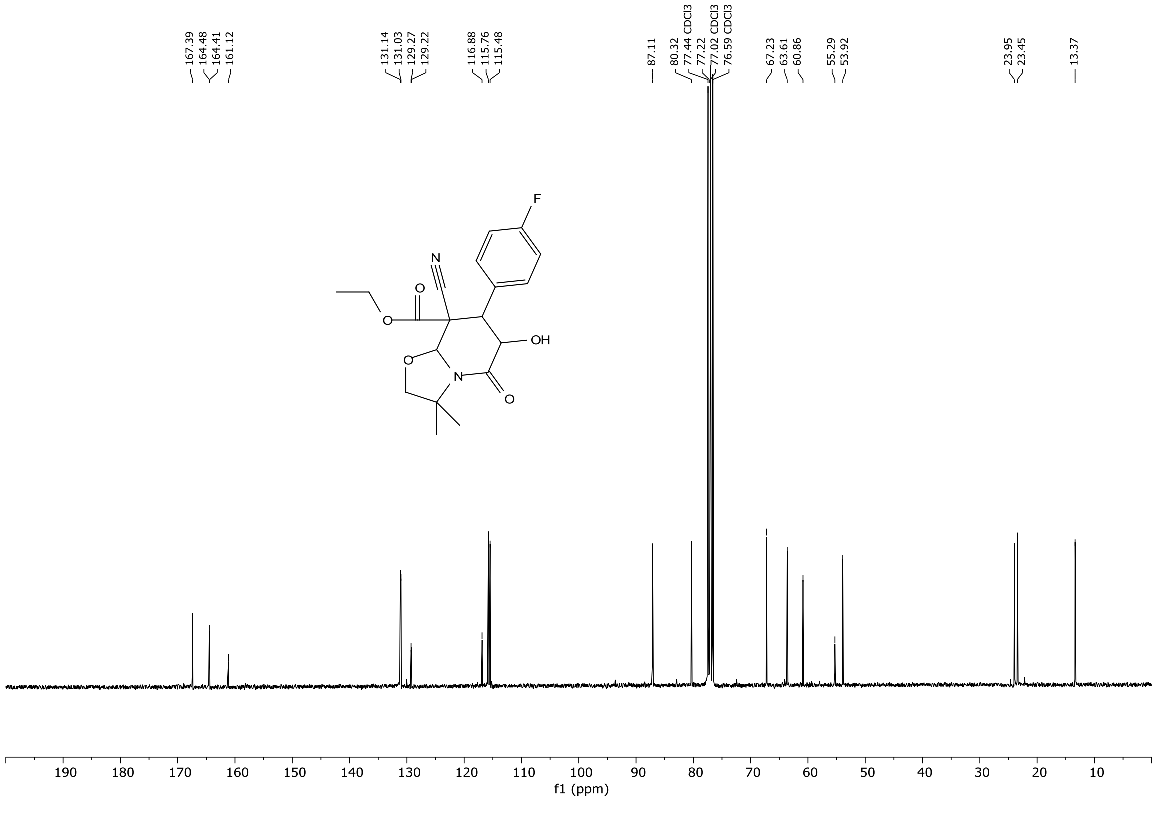

^{1}H NMR of Compound 12f (300 MHz, CDCl$_3$)

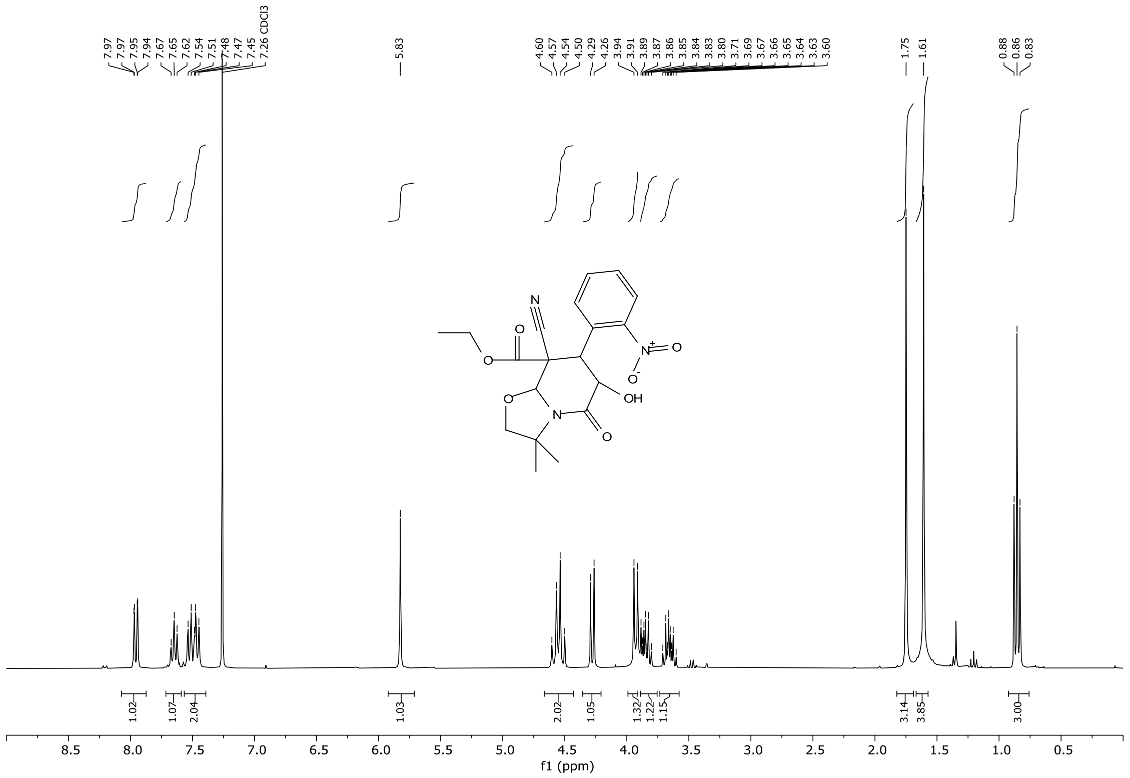

^{13}C NMR of Compound 12f (75 MHz, CDCl$_3$)

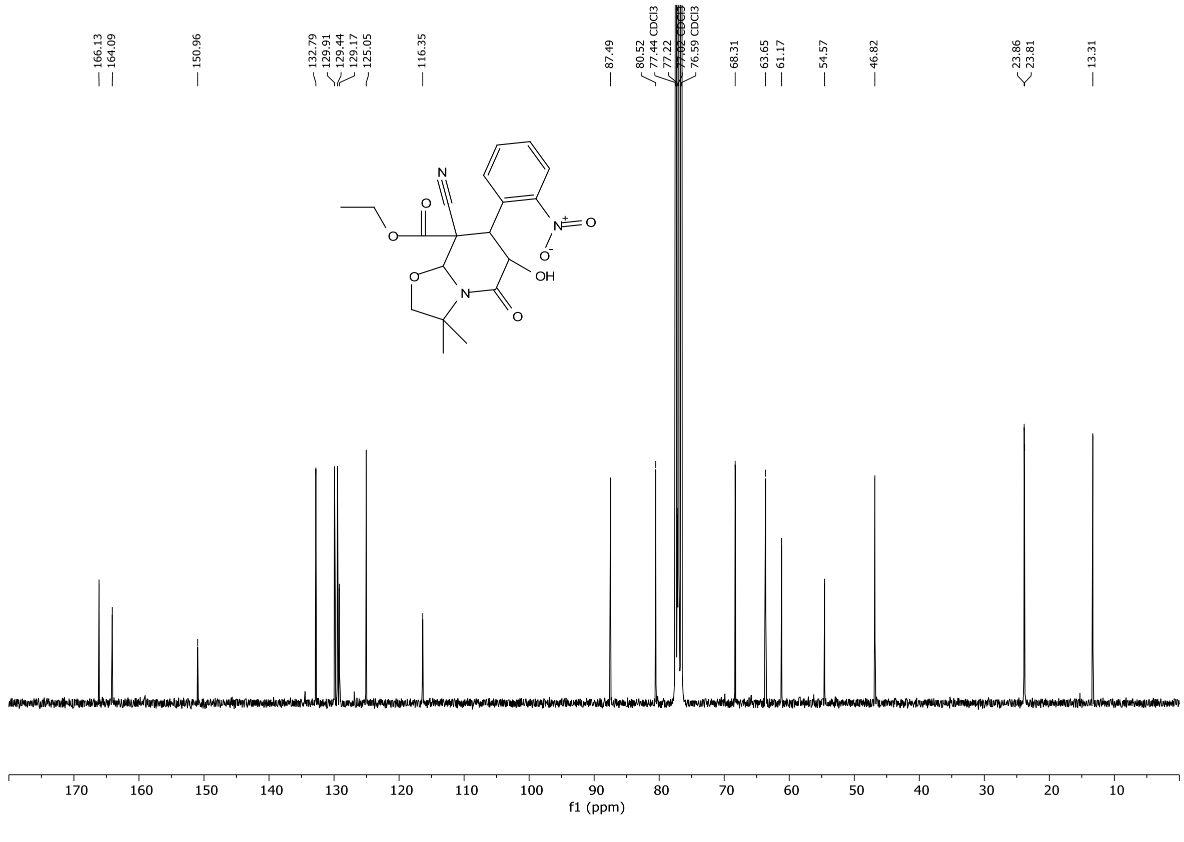

^{1}H NMR of Compound 13f (300 MHz, $CDCl_3$)

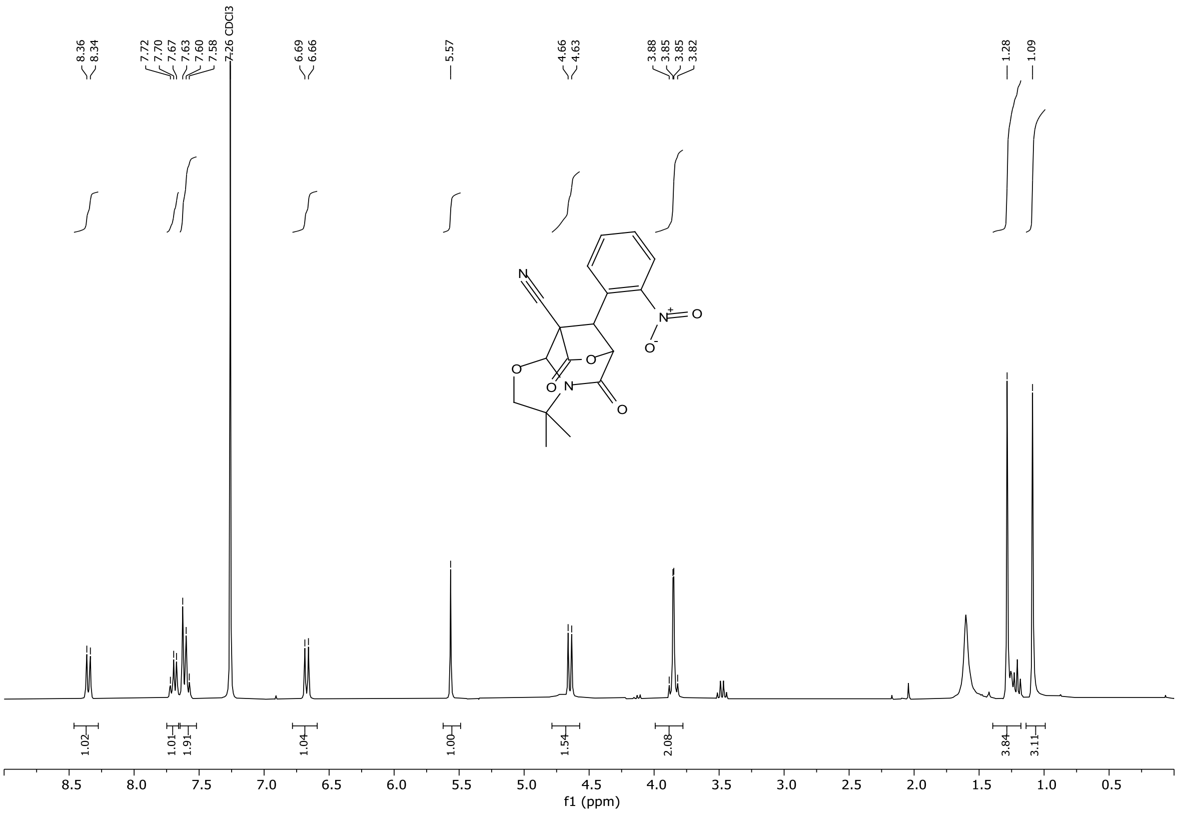

^{13}C NMR of Compound 13f (75 MHz, $CDCl_3$)

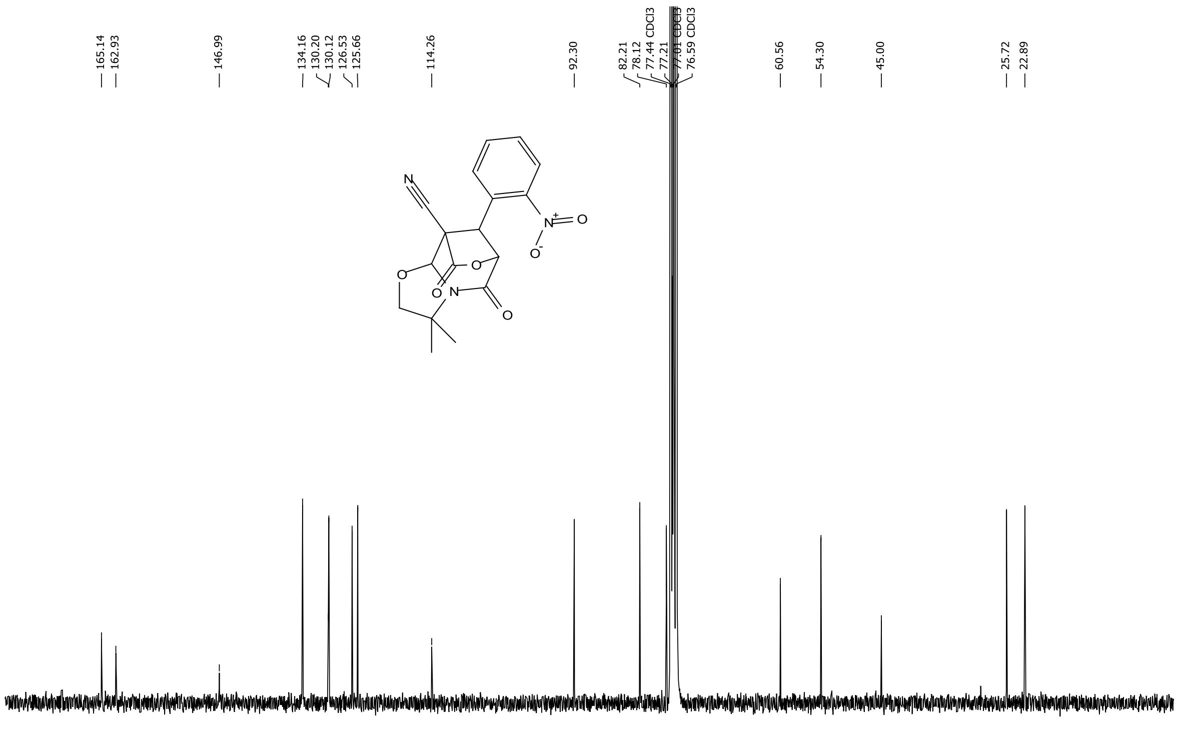

^{1}H NMR of Compound 14 (300 MHz, $CDCl_3$)

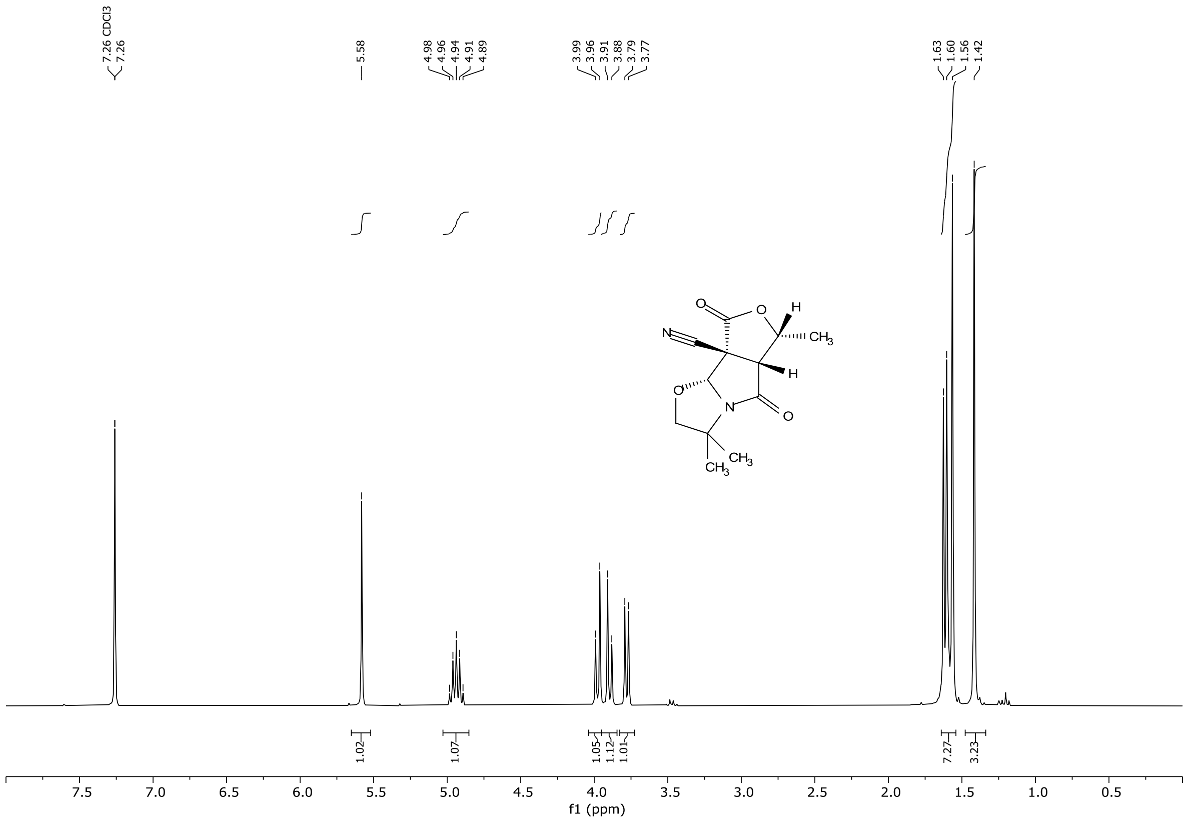

^{13}C NMR of Compound 14 (75 MHz, $CDCl_3$)

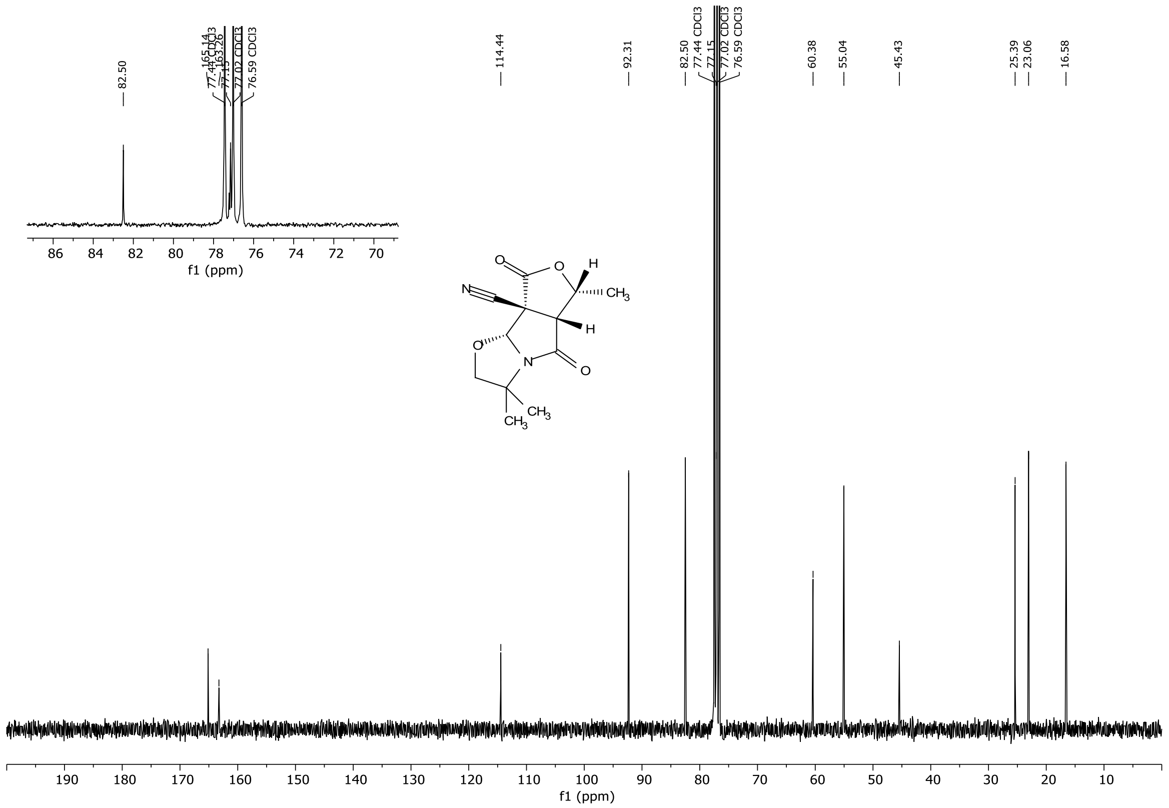

^{1}H NMR of Compound 17a (300 MHz, $CDCl_3$)

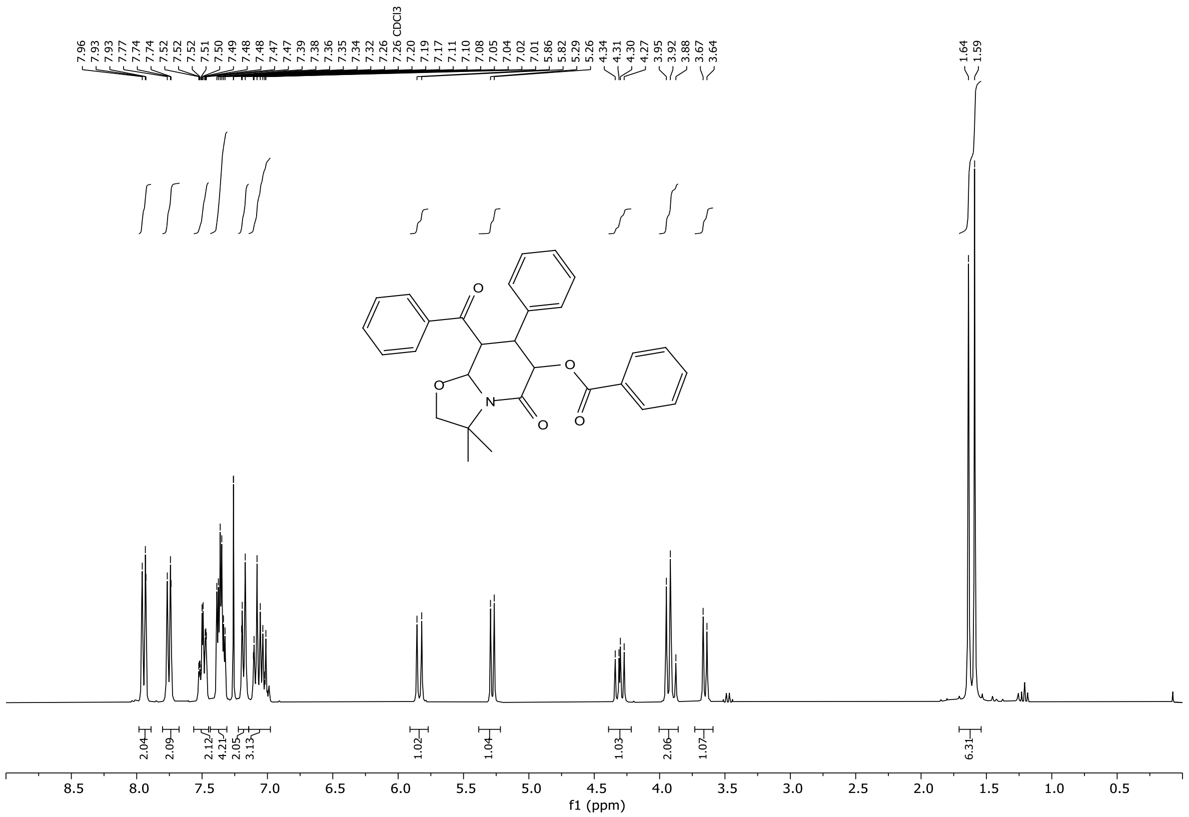

^{13}C NMR of Compound 17a (75 MHz, $CDCl_3$)

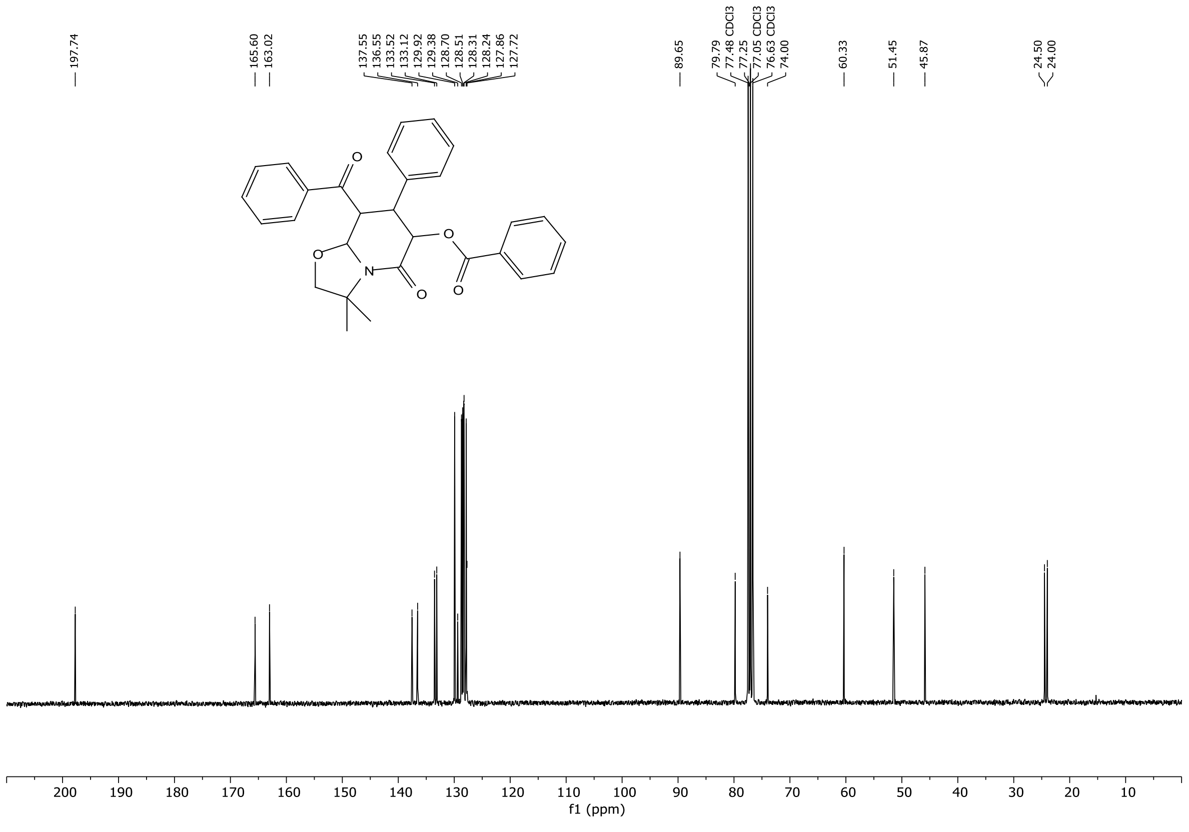

^{1}H NMR of Compound 17b (300 MHz, $CDCl_3$)

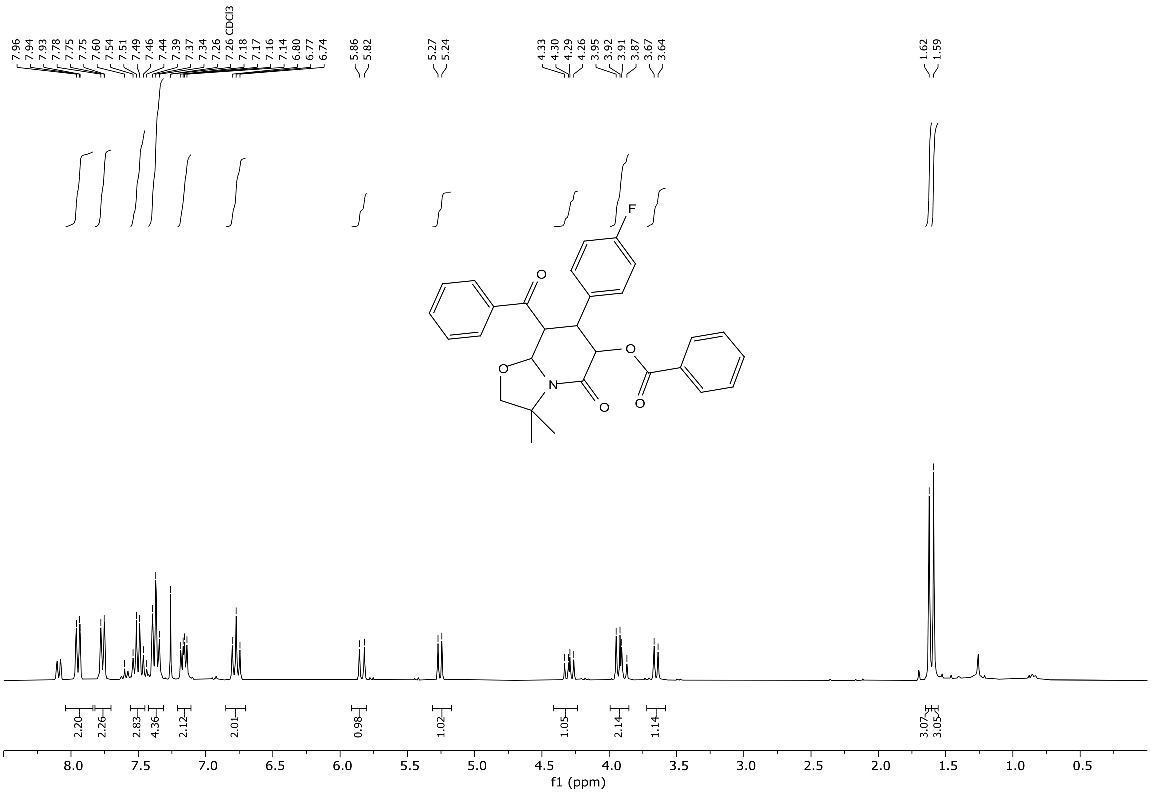

^{13}C NMR of Compound 17b (75 MHz, $CDCl_3$)

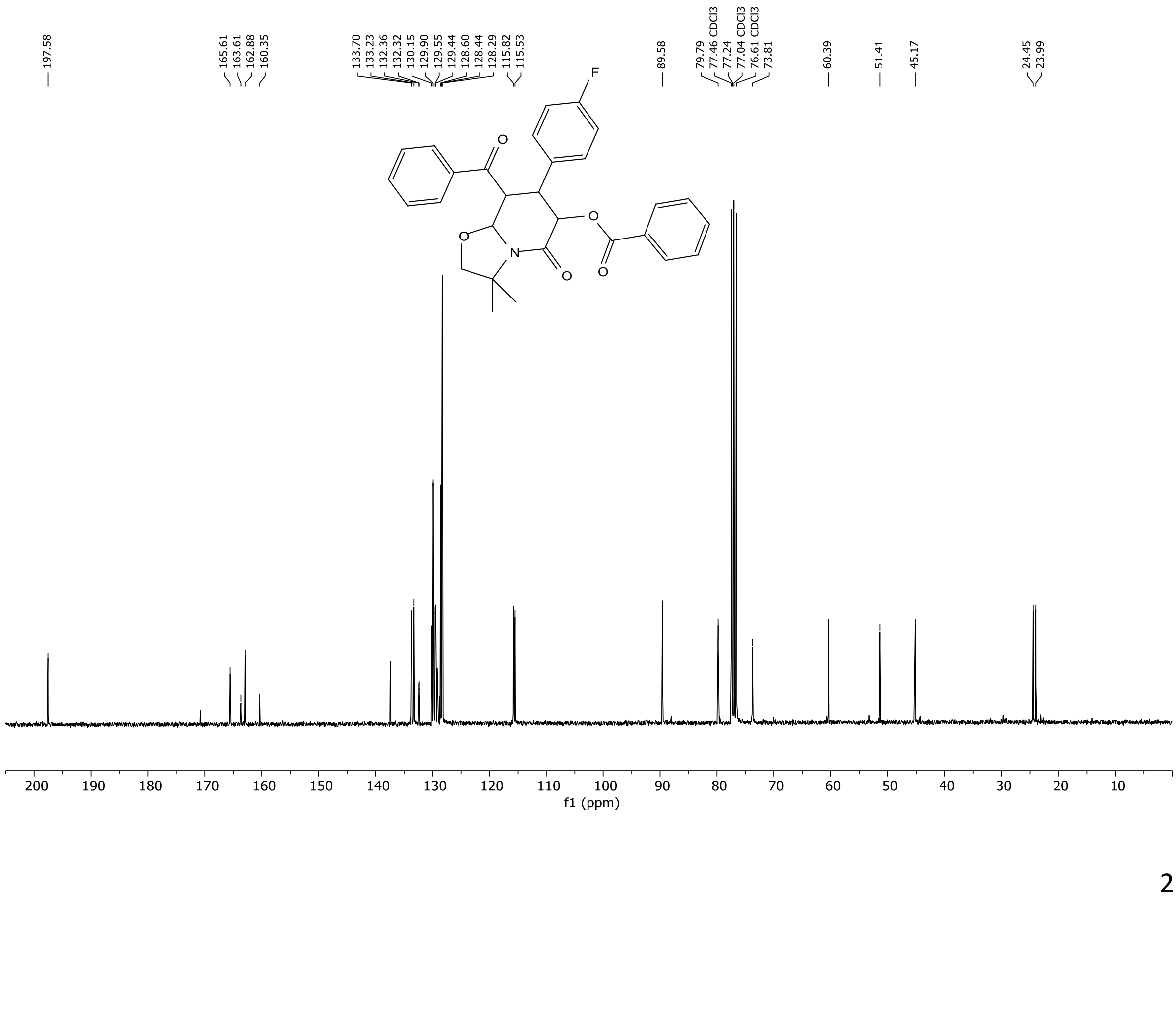

^{1}H NMR of Compound 18 (300 MHz, $CDCl_3$)

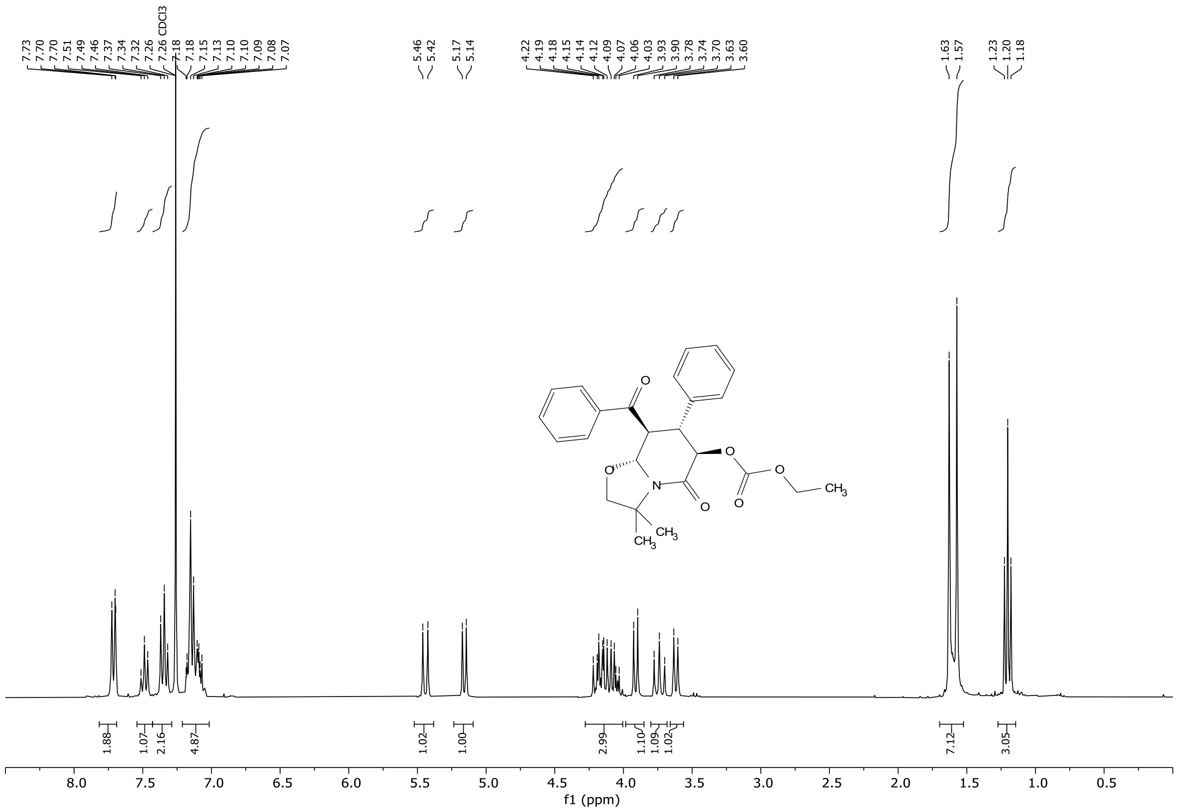

^{13}C NMR of Compound 18 (75 MHz, $CDCl_3$)

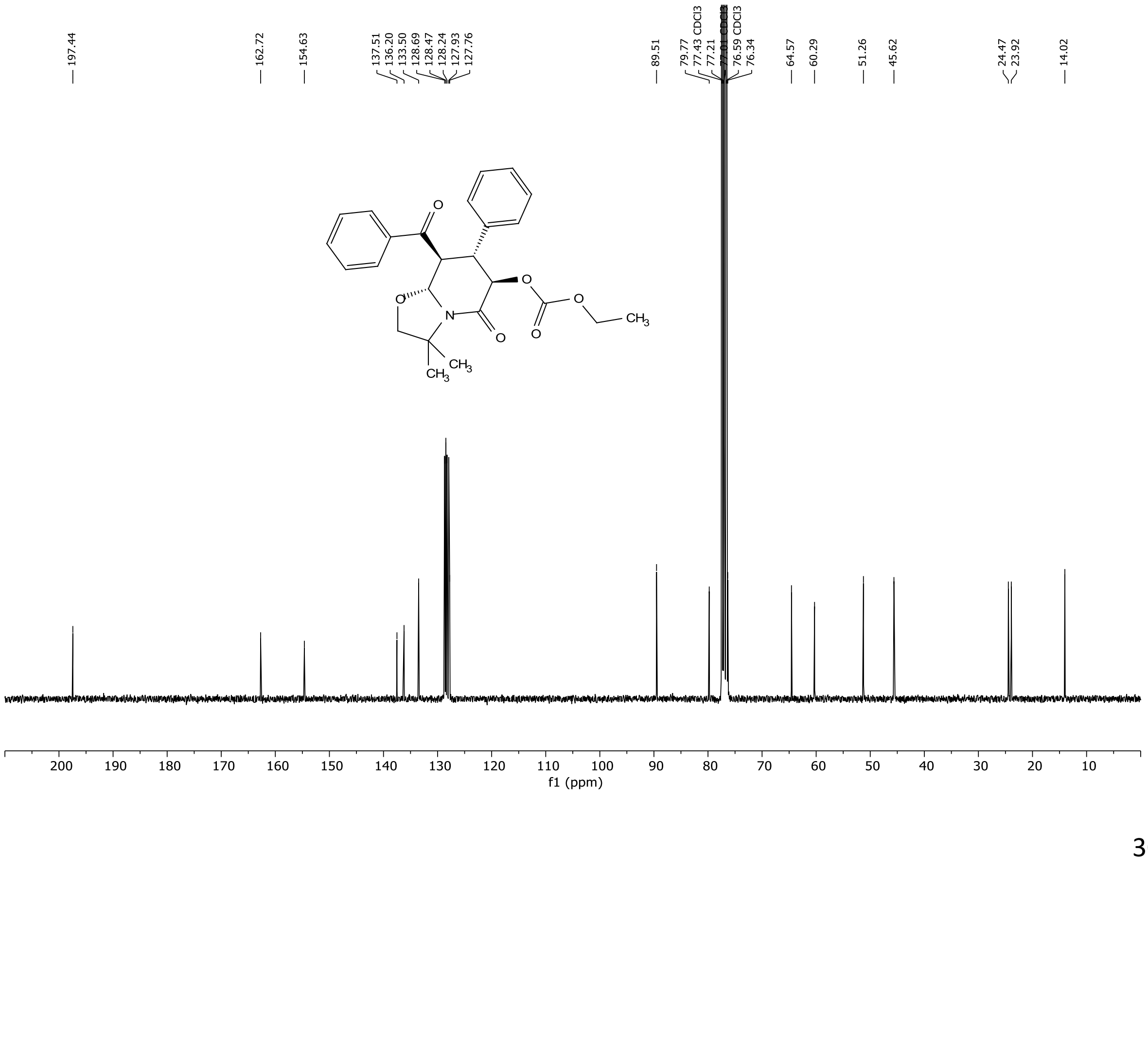

Supplement: Supplementary file 1 — Supporting Information [file OPEN-13-e202400115-s001.pdf]
